# Supplementary material for: Amazon forest resistance to drought is increased by diversity in hydraulic traits
Source: Nat Commun. 2025 Sep 9;16:8246. doi: 10.1038/s41467-025-63600-1 (PMC12420827; doi:10.1038/s41467-025-63600-1)
Supplement: Supplementary file 1 — Supplementary Information [file 41467_2025_63600_MOESM1_ESM.pdf]

Supplementary Information: Amazon forest resistance to drought is increased by diversity in hydraulic traits

Liam Langan[1*], Simon Scheiter[1], Thomas Hickler[1,2†],

Steven I. Higgins[3†].

[1] Senckenberg Biodiversity and Climate Research Centre,

Frankfurt am Main, Germany.

[2] Department of Physical Geography, Geosciences.

Goethe University, Frankfurt am Main, Germany.

[3] Plant Ecology, University of Bayreuth,

Universitätsstraße 30, Bayreuth, Germany.

[*]To whom correspondence should be addressed; E-mail: liam.langan@senckenberg.de.

[†]These authors jointly supervised this work.

Supplementary Tables and Figures

Table S1: Simulation experiment setup: Site Scale.

Spatial Scale	Forcing Data	Trait-Diversity Setup	Time Period	Forcing Variable Manipulation	Time Period Manipulation	Experiment Type	Figure Reference
Site Scale TNF	CRU RCP4.5 Clim+CO2	Full diversity Mutation + Crossover = ON	1000 years up to 2100	Daily Precipitation reduced by 50%	4 years (2000-2004)	Catastrophic Drought	Fig 1 A
Site Scale TNF	CRU RCP4.5 Clim+CO2	Varying species no.: 1, 2, ..., 96 Individuals within species have fixed traits Mutation + Crossover = OFF	1000 years up to 2100	Daily Precipitation reduced by 50%	4 years (2000-2004)	Catastrophic Drought + Diversity Level	Figs. 1 C, D, 2 A, S3, S4, S5 A
Site Scale TNF	CRU RCP4.5 Clim+CO2	Root trait variability between species removed Mutation + Crossover = OFF	1000 years up to 2100	Daily Precipitation reduced by 50%	4 years (2000-2004)	Catastrophic Drought vs No Drought	Fig. S6 A
Site Scale CAX	CRU RCP4.5 Clim+CO2	Full diversity Mutation + Crossover = ON	1000 years up to 2100	Daily Precipitation reduced by 50%	7 years (2002-2009)	Catastrophic Drought	Figs. 1 B S5 B
Site Scale CAX	CRU RCP4.5 Clim+CO2	Varying species no.: 1, 2, ..., 96 Individuals within species have fixed traits Mutation + Crossover = OFF	1000 years up to 2100	Daily Precipitation reduced by 50%	7 years (2002-2009)	Catastrophic Drought + Diversity Level	Figs. 1 C, D, 2 B, S3, S4, S5 B
Site Scale CAX	CRU RCP4.5 Clim+CO2	Root trait variability between species removed Mutation + Crossover = OFF	1000 years up to 2100	Daily Precipitation reduced by 50%	7 years (2002-2009)	Catastrophic Drought vs No Drought	Fig. S6 B

Table S2: Bayesian model regression coefficients for TNF.

**Parameter**	**Estimate**	**Est. Error**	**l-95% CI**	**u-95% CI**	**Rhat**	**Bulk ESS**	**Tail ESS**
Intercept	-30.64	0.46	-31.54	-29.73	1.00	3056	2509
sigma_Intercept	2.78	0.02	2.74	2.82	1.00	4181	2892
RAOQ	18.58	1.56	15.55	21.67	1.00	3373	2880
sigma_RAOQ	-1.46	0.10	-1.67	-1.26	1.00	3371	2773

Table S3: Bayesian model regression coefficients CAX.

**Parameter**	**Estimate**	**Est. Error**	**l-95% CI**	**u-95% CI**	**Rhat**	**Bulk ESS**	**Tail ESS**
Intercept	-39.55	0.43	-40.37	-38.71	1.00	4928	3328
sigma_Intercept	2.63	0.02	2.59	2.67	1.00	4688	2847
RAOQ	45.38	2.35	40.65	49.83	1.00	5113	2735
sigma_RAOQ	-0.31	0.12	-0.55	-0.07	1.00	4645	3031

Table S4: Number of sites for each precipitation loss interval in each RCP scenario for plant strategy removal experiment.

Number of sites for each precipitation loss interval in each RCP scenario for plant strategy removal experiment.

Precipitation	RCP 4.5			RCP 8.5		
Loss	Full Div	No Dec Wt	No Eg Wt & Dec Wt	Full Div	No Dec Wt	No Eg Wt & Dec Wt
250 - 400 (mm)	38	46	38	52	51	34
400 - 850 (mm)	122	92	74	185	140	141
>850 (mm)	29	46	38	83	93	75

Table S5: Simulation experiment setup: Continental Scale.

Spatial Scale	Forcing Data	Trait-Diversity Setup Plant Strategy Removal (PS)	Time Period	Forcing Variable Manipulation	Time Period Manipulation	Experiment Type	Figure Reference
Continental Scale	CRU RCP4.5 Clim+CO2	Full diversity Mutation + Crossover = ON	1000 years up to 2100	Precipitation - RCP anomaly Temperature - RCP anomaly CO2 - following RCP	2000 - 2100	Chronic Climate Change	Figs. 3 A, 4 A, S7 A, S8, S9, S10, S11 A, S12 A, S13 A, C, E
Continental Scale	CRU RCP4.5 Clim+CO2	PS - Deciduous water triggered phenology Mutation + Crossover = ON	1000 years up to 2100	Precipitation - RCP anomaly Temperature - RCP anomaly CO2 - following RCP	2000 - 2100	Chronic Climate Change + Diversity	Figs. 4 A, S11 B, S12 A, S13 A, C, E
Continental Scale	CRU RCP4.5 Clim+CO2	PS - Deciduous and evergreen water triggered phenology Mutation + Crossover = ON	1000 years up to 2100	Precipitation - RCP anomaly Temperature - RCP anomaly CO2 - following RCP	2000 - 2100	Chronic Climate Change + Diversity	Figs. 4 A, S11 C, S12 A, S13 A, C, E
Continental Scale	CRU RCP4.5 Clim	Full diversity Mutation + Crossover = ON	1000 years up to 2100	Precipitation - RCP anomaly Temperature - RCP anomaly CO2 - fixed at 1990 level	2000 - 2100	Chronic Climate Change	Figs. 3 B, 4 C, S7 B, S8, S12 C, S13 B, D, F
Continental Scale	CRU RCP4.5 Clim	PS - Deciduous water triggered phenology Mutation + Crossover = ON	1000 years up to 2100	Precipitation - RCP anomaly Temperature - RCP anomaly CO2 - fixed at 1990 level	2000 - 2100	Chronic Climate Change + Diversity	Figs. 4 C, S12 C, S13 B, D, F
Continental Scale	CRU RCP4.5 Clim	PS - Deciduous and evergreen water triggered phenology Mutation + Crossover = ON	1000 years up to 2100	Precipitation - RCP anomaly Temperature - RCP anomaly CO2 - fixed at 1990 level	2000 - 2100	Chronic Climate Change + Diversity	Figs. 4 C, S12 C, S13 B, D, F
Continental Scale	CRU RCP8.5 Clim+CO2	Full diversity Mutation + Crossover = ON	1000 years up to 2100	Precipitation - RCP anomaly Temperature - RCP anomaly CO2 - following RCP	2000 - 2100	Chronic Climate Change	Figs. 3 C, 4 B, S7 C, S8, S12 B, S13 A, C, E
Continental Scale	CRU RCP8.5 Clim+CO2	PS - Deciduous water triggered phenology Mutation + Crossover = ON	1000 years up to 2100	Precipitation - RCP anomaly Temperature - RCP anomaly CO2 - following RCP	2000 - 2100	Chronic Climate Change + Diversity	Figs. 4 B, S12 B, S13 A, C, E
Continental Scale	CRU RCP8.5 Clim+CO2	PS - Deciduous and evergreen water triggered phenology Mutation + Crossover = ON	1000 years up to 2100	Precipitation - RCP anomaly Temperature - RCP anomaly CO2 - following RCP	2000 - 2100	Chronic Climate Change + Diversity	Figs. 4 B, S12 B, S13 A, C, E
Continental Scale	CRU RCP8.5 Clim	Full diversity Mutation + Crossover = ON	1000 years up to 2100	Precipitation - RCP anomaly Temperature - RCP anomaly CO2 - fixed at 1990 level	2000 - 2100	Chronic Climate Change	Figs. 3 D, 4 D, S7 D, S8, S12 D, S13 B, D, F
Continental Scale	CRU RCP8.5 Clim	PS - Deciduous water triggered phenology Mutation + Crossover = ON	1000 years up to 2100	Precipitation - RCP anomaly Temperature - RCP anomaly CO2 - fixed at 1990 level	2000 - 2100	Chronic Climate Change + Diversity	Figs. 4 D, S12 D, S13 B, D, F
Continental Scale	CRU RCP8.5 Clim	PS - Deciduous and evergreen water triggered phenology Mutation + Crossover = ON	1000 years up to 2100	Precipitation - RCP anomaly Temperature - RCP anomaly CO2 - fixed at 1990 level	2000 - 2100	Chronic Climate Change + Diversity	Figs. 4 D, S12 D, S13 B, D, F

Table S6: Summary of linear mixed-effects model used to assess the interactive effects of vegetation stand properties, precipitation changes, RCP scenario, increasing or fixed CO_2, and functional diversity on the percentage change in above ground biomass between 1990 and 2100. Continuous predictor variables were scaled prior to analysis, estimates at therefore standardised. Fig. 4 displays predicted relationships between functional diversity, precipitation change, and CO_2 on changes in future biomass. No correction for multiple comparisons was applied.

	Predictors	Estimates	CI	p
1	(Intercept)	-10.88	-12.60 – -9.17	<0.001
2	Precipitation 1990	8.18	6.53 – 9.82	<0.001
3	RaoQ	1.73	0.33 – 3.13	0.015
4	Precipitation Change	-10.40	-11.92 – -8.87	<0.001
5	CO2 [NOCO2]	-27.12	-29.07 – -25.18	<0.001
6	RCP [8.5]	12.13	10.28 – 13.97	<0.001
7	Precipitation 1990 × RaoQ	-1.72	-3.13 – -0.30	0.017
8	Precipitation 1990 × Precipitation Change	1.40	-0.31 – 3.10	0.108
9	RaoQ × Precipitation Change	0.99	-0.34 – 2.31	0.143
10	Precipitation 1990 × CO2[NOCO2]	-1.41	-3.28 – 0.47	0.142
11	RaoQ × CO2 [NOCO2]	-0.26	-2.05 – 1.54	0.779
12	Precipitation Change × CO2[NOCO2]	1.02	-0.83 – 2.87	0.280
13	Precipitation 1990 × RCP[8.5]	1.18	-0.59 – 2.96	0.191
14	RaoQ × RCP[8.5]	-0.82	-2.54 – 0.91	0.354
15	Precipitation Change × RCP[8.5]	-0.64	-2.44 – 1.16	0.484
16	CO2[NOCO2] × RCP[8.5]	-26.85	-29.25 – -24.45	<0.001
17	(Precipitation 1990 × RaoQ) × Precipitation Change	-0.15	-1.70 – 1.40	0.849
18	(Precipitation 1990 × RaoQ) × CO2[NOCO2]	1.22	-0.60 – 3.03	0.189
19	(Precipitation 1990 × Precipitation Change) × CO2[NOCO2]	-0.53	-2.61 – 1.55	0.618
20	(RaoQ × Precipitation Change) × CO2[NOCO2]	0.57	-1.16 – 2.31	0.518
21	(Precipitation 1990 × RaoQ) × RCP[8.5]	0.59	-1.17 – 2.35	0.512
22	(Precipitation 1990 × Precipitation Change) × RCP[8.5]	2.40	0.49 – 4.31	0.014
23	(RaoQ × Precipitation Change) × RCP[8.5]	0.11	-1.61 – 1.83	0.900
24	(Precipitation 1990 × CO2[NOCO2]) × RCP[8.5]	-2.82	-5.17 – -0.47	0.019
25	(RaoQ × CO2[NOCO2]) × RCP[8.5]	0.88	-1.47 – 3.23	0.462
26	(Precipitation Change × CO2[NOCO2]) × RCP[8.5]	2.63	0.27 – 4.99	0.029
27	(Precipitation 1990 × RaoQ × Precipitation Change) × CO2[NOCO2]	0.00	-2.01 – 2.01	0.999
28	(Precipitation 1990 × RaoQ × Precipitation Change) × RCP[8.5]	-1.17	-3.06 – 0.72	0.225
29	(Precipitation 1990 × RaoQ × CO2[NOCO2]) × RCP[8.5]	-0.48	-2.88 – 1.92	0.695
30	(Precipitation 1990 × Precipitation Change × CO2[NOCO2]) × RCP[8.5]	0.18	-2.32 – 2.68	0.888
31	(RaoQ × Precipitation Change × CO2[NOCO2]) × RCP[8.5]	0.97	-1.35 – 3.28	0.414
32	(Precipitation 1990 × RaoQ × Precipitation Change × CO2[NOCO2]) × RCP [8.5]	0.62	-1.91 – 3.15	0.630
33	Random Effects			
34	$\sigma 2$	395.47		
35	$\tau 00 Site$	130.89		
36	ICC	0.25		
37	N Site	847		
38	Observations	5394		
39	Marginal R2 / Conditional R2	0.556 / 0.666		
40	AIC	48477.427		

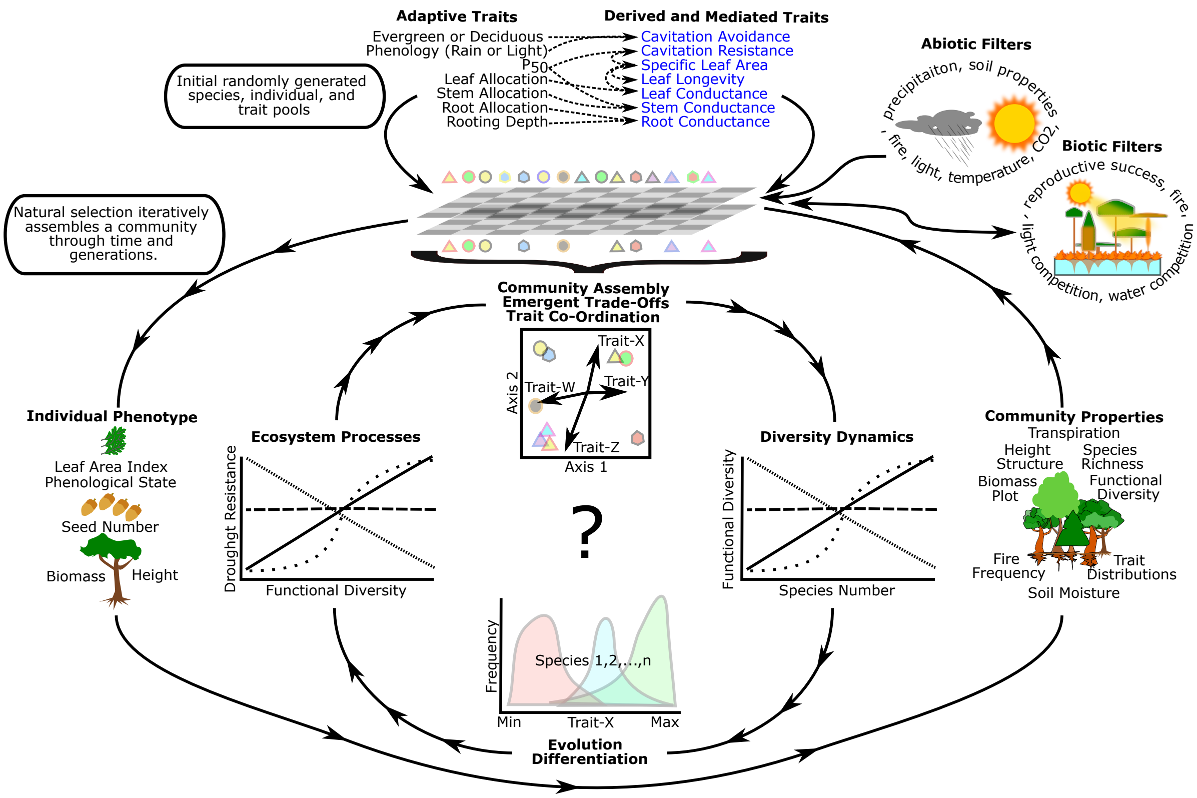

Figure S1: **aDGVM2-Pi conceptual framework - modelling relationships between biodiversity and ecosystem function**: Starting from an initial population of individuals with randomly chosen trait values, the process of natural selection iteratively forms a community through time and generations. Changing abiotic and biotic conditions leads to the filtering of individuals whose trait composition and phenotypic state result in low relative fitness. Resultant emergent dynamics allow investigation of ecosystem processes and how these my be affected by community characteristics.

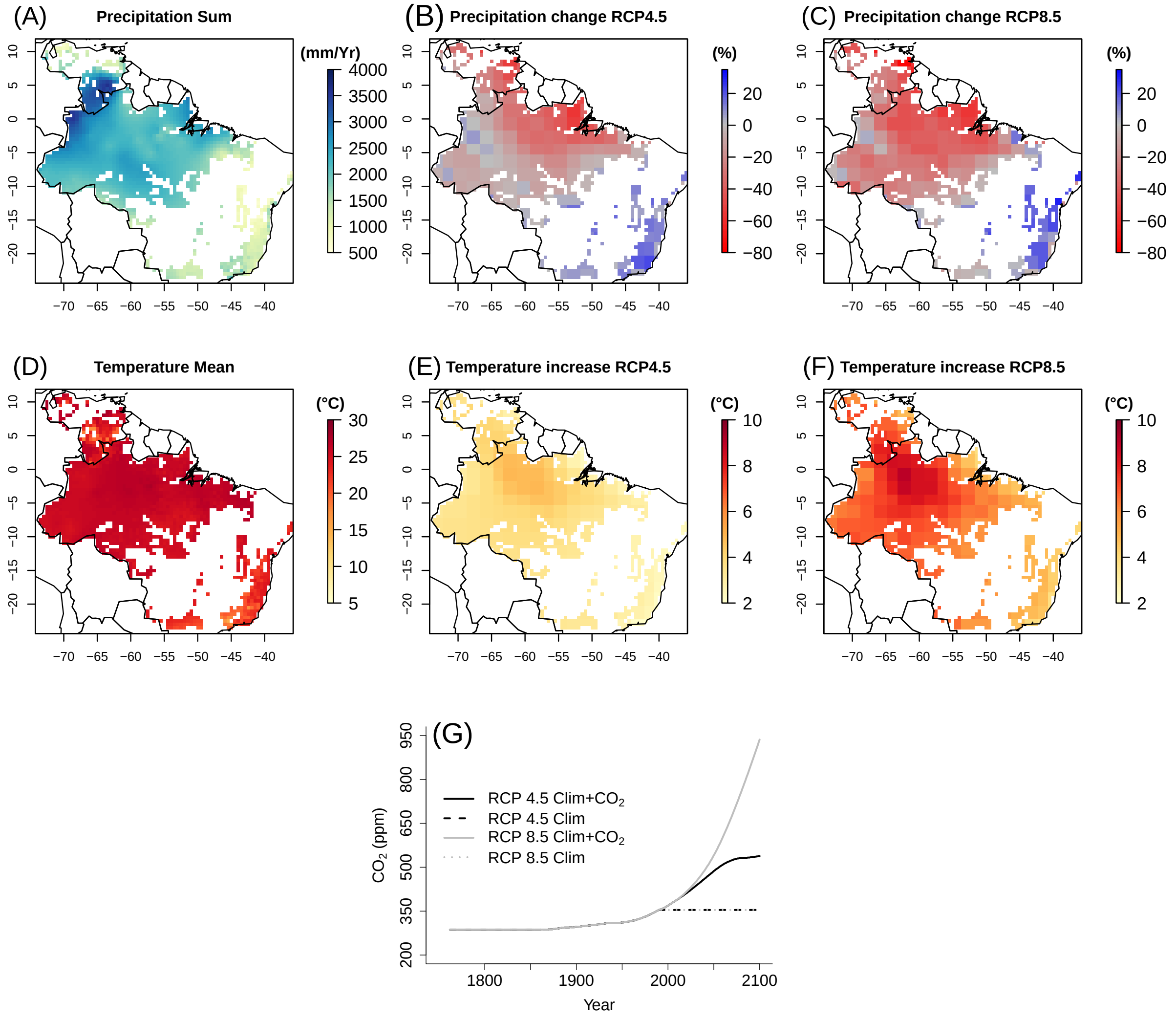

Figure S2: **Precipitation, temperature, and CO_2 forcing input and applied RCP anomalies**: (A) Mean annual Precipitation for the period 1961-1990 (B) Precipitation change in 2100 for RCP4.5, (C) Precipitation change in 2100 for RCP8.5, (D) mean annual temperature for the period 1961-1990, (E) Temperature change in 2100 for RCP4.5, (F) Temperature change in 2100 for RCP8.5, (G) CO2 change per RCP scenario. A and C are derived from CRU[1] data. CO2 concentrations were increased inline with combined historical and future change data[2] or held fixed (G), precipitation, and temperature anomalies were created using combined historical (1850-2005) and future change (RCP data 2006-2100) from the MPI-ESM-LR ESM.[2] Anomalies were centered (zero change) on the period 2001-2010 to smooth transitions between historical and future change data. Savanna sites were removed using the observed distribution from Lehmann et al.[3] The data underlying this figure are provided in figshare (https://doi.org/10.6084/m9.figshare.26232395).

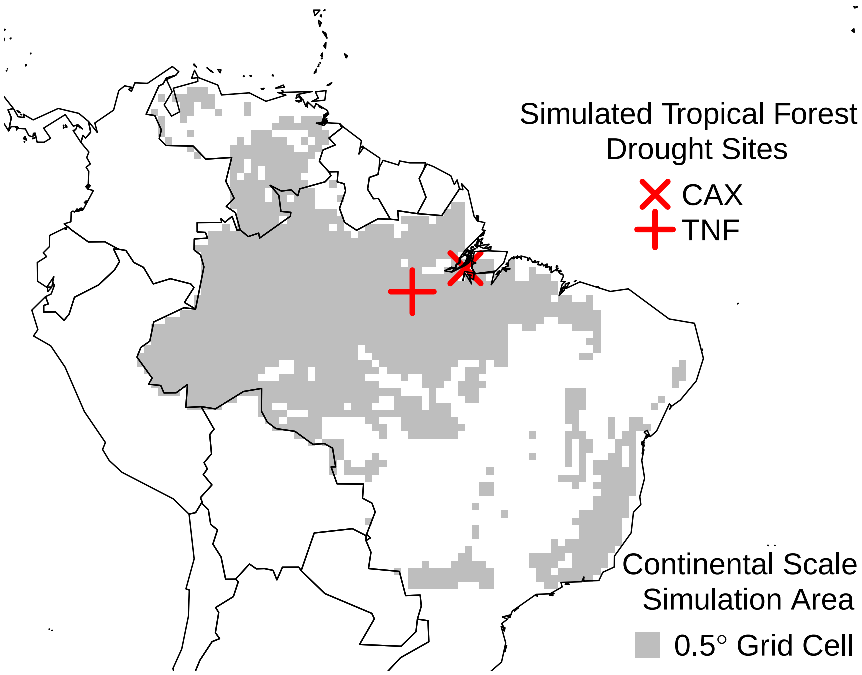

Figure S3: **South American Study Region**: red symbols indicate the position of two simulated drought sites in Tapajos (TNF) and Caxiuana (CAX) National Forests. Grey symbol indicates simulated tropical forest grid cells in Brazil and Venezuela, north of 23° south, and below 1000m, for future change simulations. Savanna sites were removed using the observed distribution from Lehmann et al.[3]

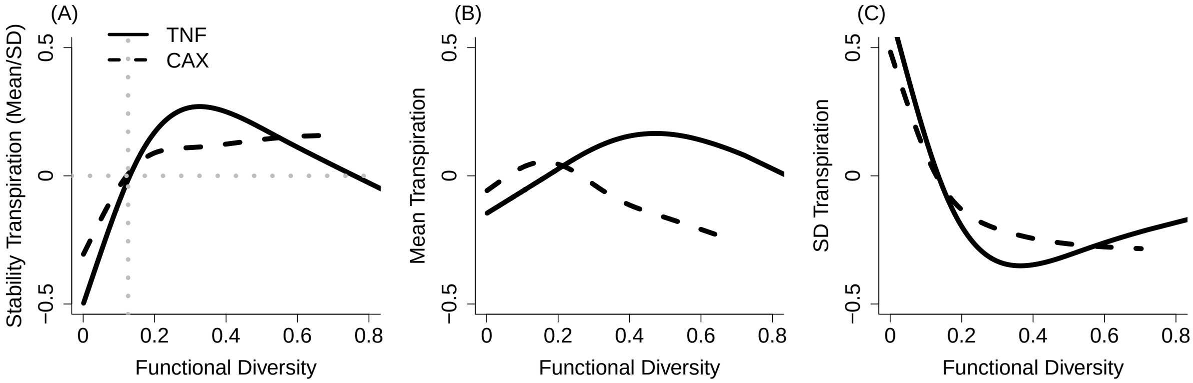

Figure S4: **Transpiration stability**: (A) shows the normalised stability of transpiration plotted against functional diversity. Stability is calculated as the mean divided by the standard deviation of transpiration. (B) shows the normalised mean transpiration. (C) shows the normalised standard deviation. The mean was calculated as the mean of the average daily transpiration in the 50 years prior to drought. The standard deviation is the mean of the within year standard deviation in daily transpiration in the 50 years prior to drought. All panels display spline regressions with three knots. The horizontal line in (A) displays the zero line. The vertical gray line in (A) shows the value of functional diversity (mean of TNF and CAX) where the zero line is crossed. The mean was chosen as separate lines were too close to distinguish visually. Functional diversity (RaoQ) was calculated using the 8 traits shown in Fig. 2. The data underlying this figure are provided in figshare (https://doi.org/10.6084/m9.figshare.26232395).

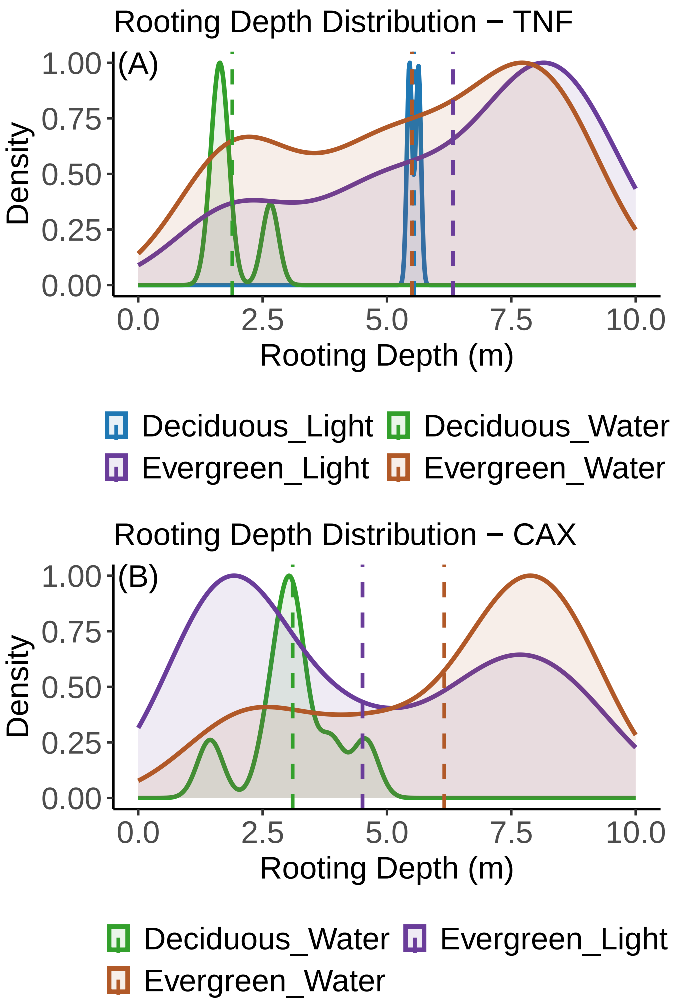

Figure S5: **Root niche differentiation**: Density plot of the distribution of rooting depths for plant strategies at Tapajos National Forest site (TNF) (A) and Caxiuana Natyional Forest site (CAX) (B). Plant strategies are, deciduous trees with a leaf phenological trigger which responds to changes in light (Deciduous_Light), deciduous trees with a leaf phenological trigger which responds to changes in soil water (Deciduous_Water), evergreen trees with a phenological trigger which responds to changes in light (Evergreen_Light), and evergreen trees with a leaf phenological trigger which responds to changes in soil water (Evergreen_Water). Densities were scaled to a maximum value of one. Colours correspond to the colours of plant strategies in Fig. 2 with densities calculated for each site and plant strategy using the same 96 species. Vertical lines indicate the mean of the density distribution for each plant strategy. The data underlying this figure are provided in figshare (https://doi.org/10.6084/m9.figshare.26232395).

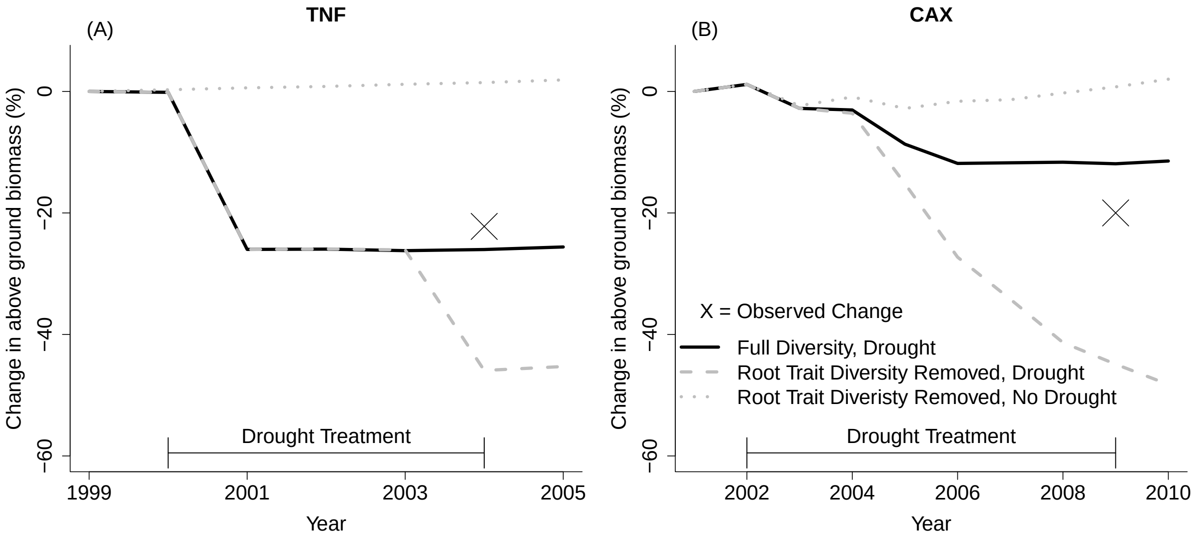

Figure S6: **Removing root niche differentiation**: Shown are drought simulations run for TNF (A) and CAX (B) where diversity in below-ground rooting niches (Fig. S5) was removed. Black lines show model response to drought with no changes to traits. Dashed lines show drought response for simulations in which diversity in rooting traits was removed on the first day of drought. Dotted lines are a control showing the affect of removing diversity in rooting traits without drought. Communities were identical in traits and state variables (biomass, tree height, etc.) before drought. Below ground traits (rooting depth, root par 1, root par 2 (see tab A3)) were set to the mean values of the deepest rooting strategy at the CAX site (Evergreen_Water, Fig. S5). To aid comparability of treatments the same values were used for both sites. The data underlying this figure are provided in figshare (https://doi.org/10.6084/m9.figshare.26232395).

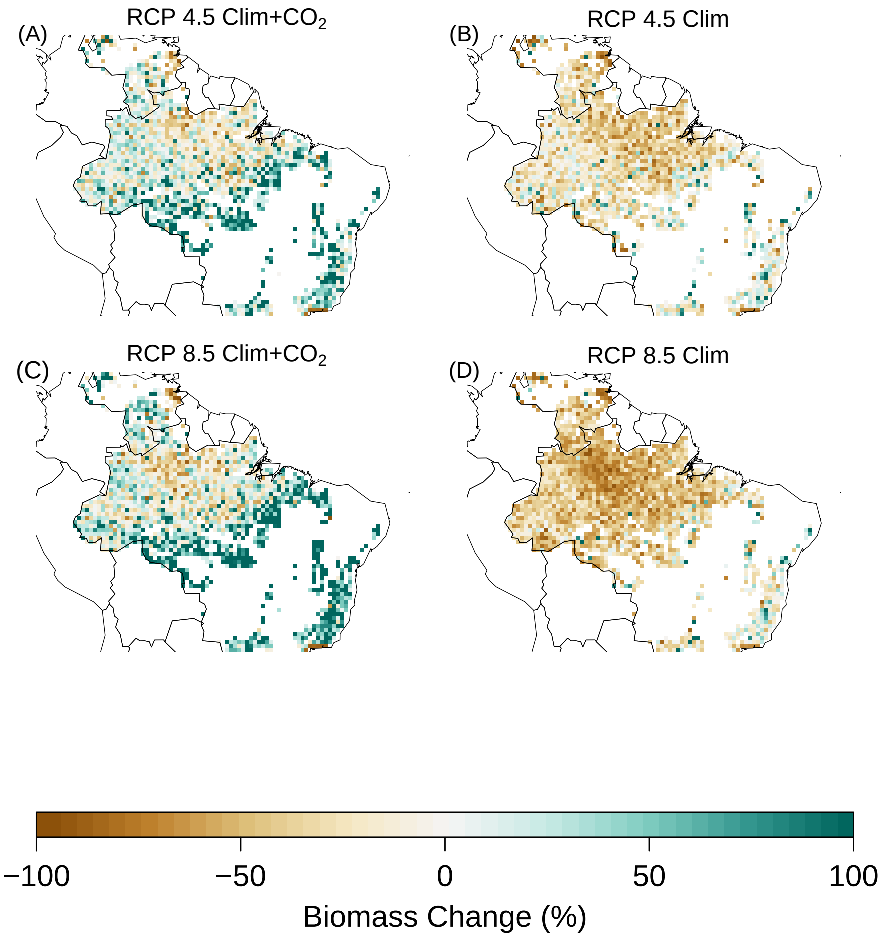

Figure S7: **Amazonian future biomass change:** Maps show the spatial distribution of biomass changes in the year 2100. (A) RCP 4.5 with increasing CO_2, (B) RCP 4.5 with fixed CO_2, (C) RCP 8.5 with increasing CO_2, (D) RCP 8.5 with fixed CO_2. Percentage changes greater than 100% were set to 100%. Savanna sites were removed using the observed distribution from Lehmann et al.[3] The data underlying this figure are provided in figshare (https://doi.org/10.6084/m9.figshare.26232395).

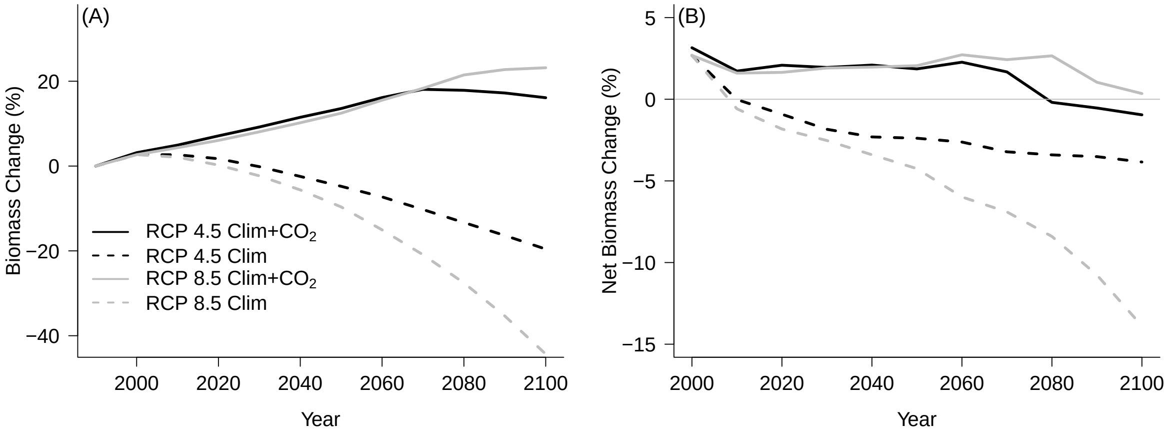

Figure S8: **Amazonian biomass change between 1990-2100:** (A) Percentage change in the total biomass stored in the study region. (B) Decadal net biomass change (%). In B, values above the horizontal line at zero indicate a net biomass gain (SINK) while values below the horizontal line at zero indicate a net biomass loss (SOURCE). The data underlying this figure are provided in figshare (https://doi.org/10.6084/m9.figshare.26232395).

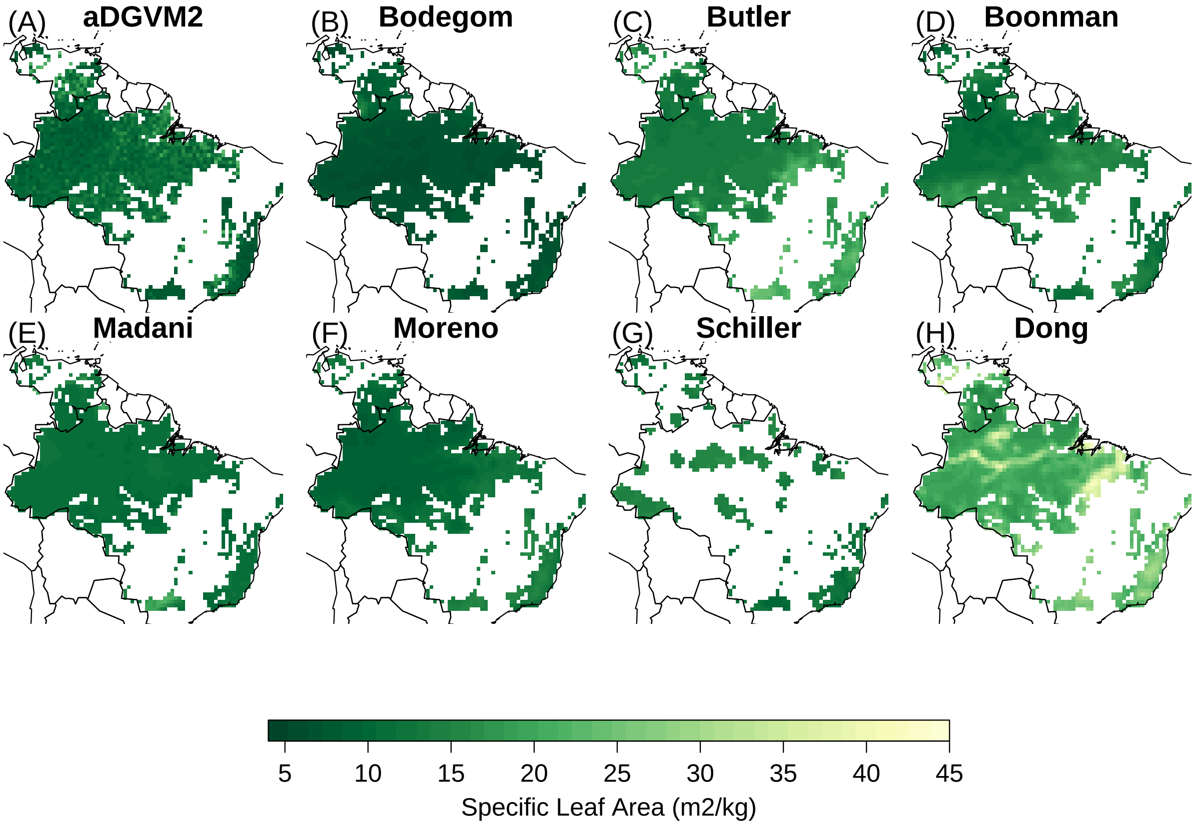

Figure S9: **Specific leaf area spatial distribution**: Specific leaf area (SLA) distribution for the study region derived from: (A) aDGVM2 simulations; (B) van Bodegom et al.;[4] (C) Butler et al.;[5] (D) Boonman et al.;[6] (E) Mandani et al.;[7] (F) Moreno et al.;[8] (G) Schiller et al.;[9] (H) Dong et al.[10] Data above (B-F) were downloaded from the supplementary data of Dong et al.[10] at half degree resolution. Savanna sites were removed using the observed distribution from Lehmann et al.[3] The data underlying this figure are provided in figshare (https://doi.org/10.6084/m9.figshare.26232395).

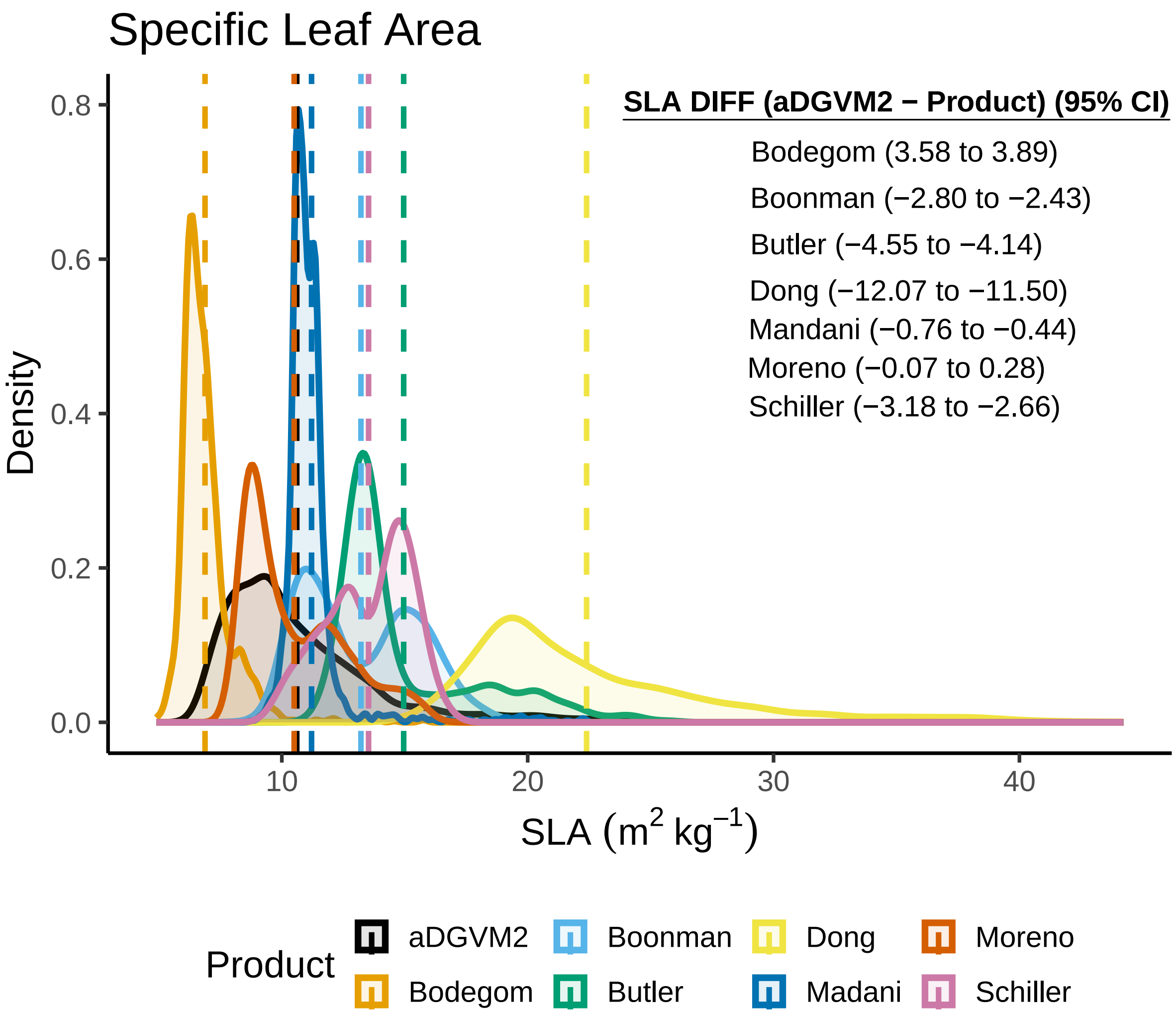

Figure S10: **Specific leaf area for the Amazon region**: Density plot and comparison of SLA predictions for the study region derived from: aDGVM2 simulations; van Bodegom et al.;[4] Butler et al.;[5] Boonman et al.;[6] Mandani et al.;[7] Moreno et al.;[8] Schiller et al.;[9] Dong et al.[10] Non-aDGVM2 data were downloaded from the supplementary data of Dong et al.[10] at half degree resolution. The data underlying this figure are provided in figshare (https://doi.org/10.6084/m9.figshare.26232395).

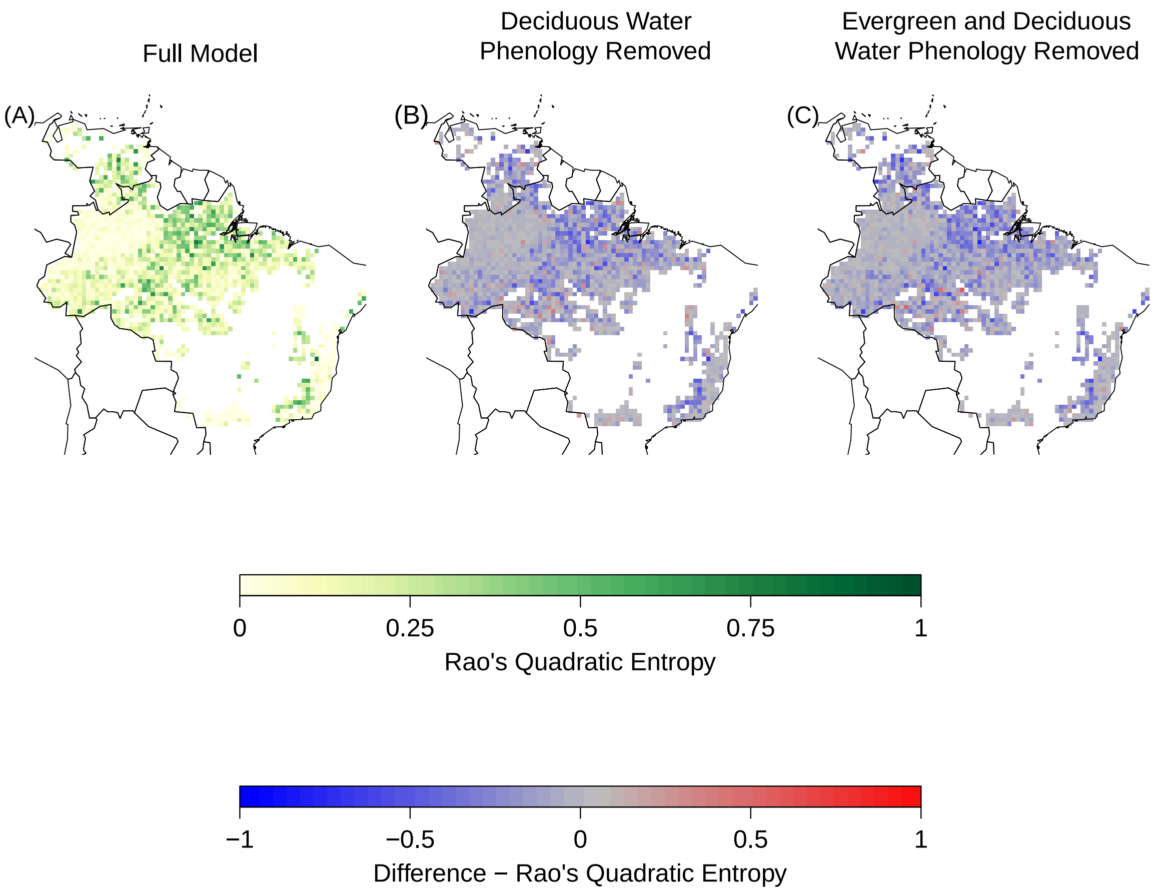

Figure S11: **Functional diversity and changes caused by plant strategy removal:** A) Functional diversity for the study area calculated as Rao's quadratic entropy. B) Reductions in functional diversity when deciduous plants with a phenological trigger that responds to soil water are removed. C) Reductions in functional diversity when deciduous and evergreen plants with a phenological trigger that responds to soil water are removed. Functional diversity was calculated as Rao's quadratic entropy. We used the hydraulic traits, P50 and maximum rooting depth, to calculate FD as these were not directly manipulated by removing phenological strategies. Savanna sites were removed using the observed distribution from Lehmann et al.[3] The data underlying this figure are provided in figshare (https://doi.org/10.6084/m9.figshare.26232395).

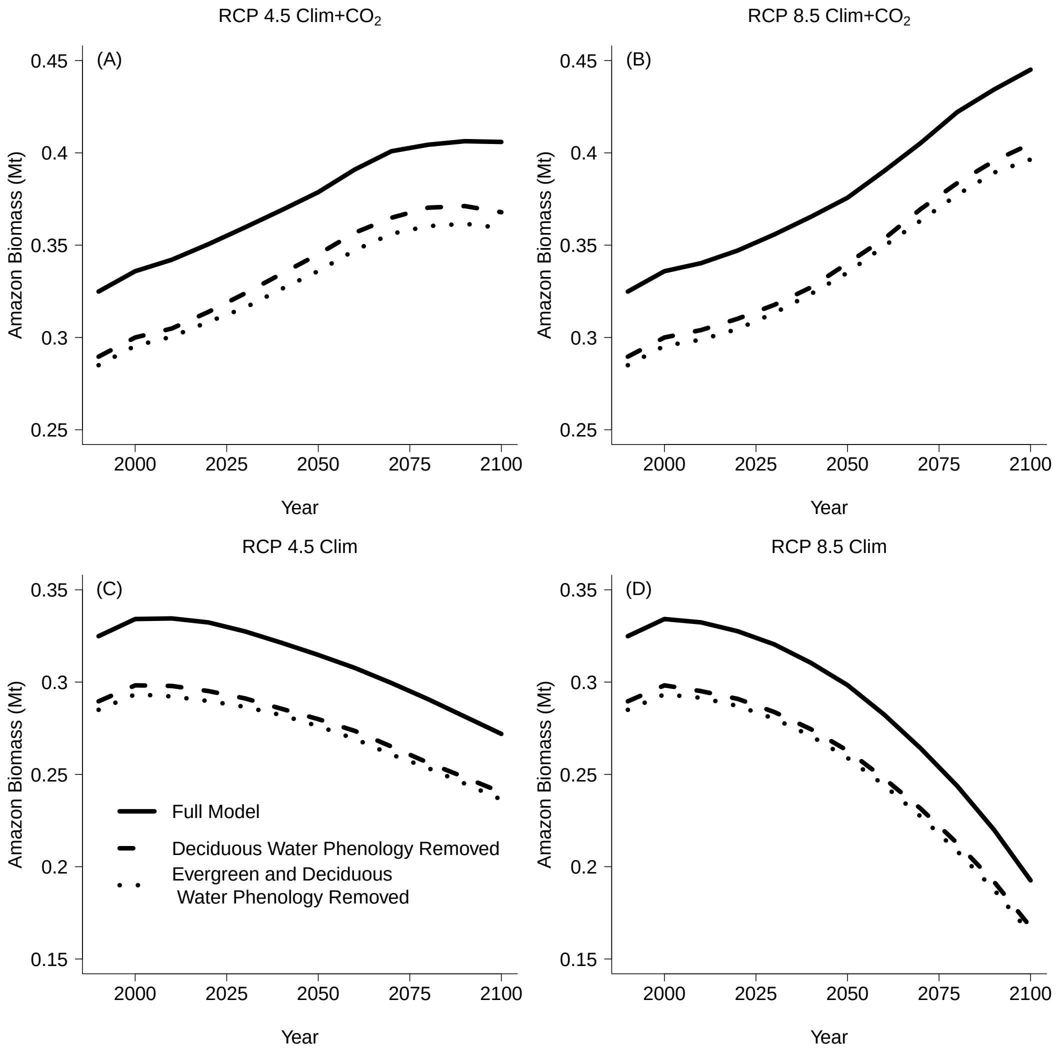

Figure S12: **Study area biomass trajectories with removal of plant strategies**: (A) RCP 4.5 climate forcing and increasing CO_2, (B) RCP 8.5 climate forcing and increasing CO_2, (C) RCP 4.5 climate forcing with fixed CO_2, (D) RCP 8.5 climate forcing with fixed CO_2. The data underlying this figure are provided in figshare (https://doi.org/10.6084/m9.figshare.26232395).

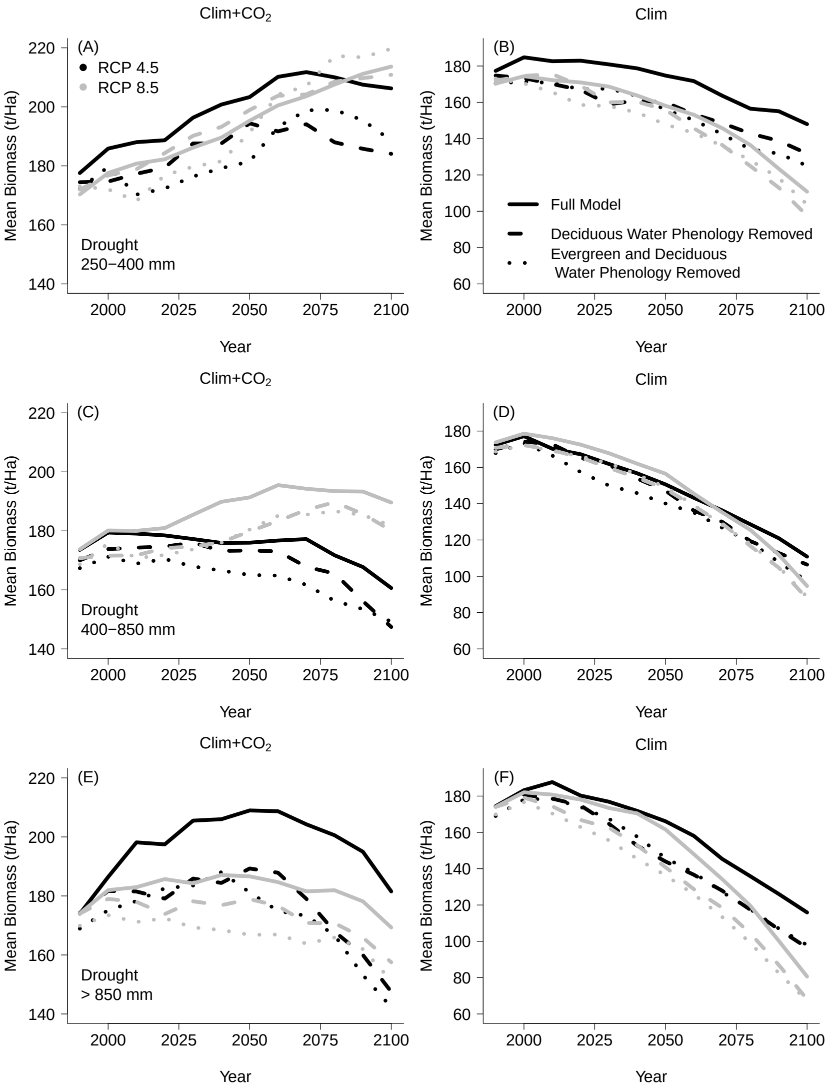

Figure S13: **Mean biomass for sites in each precipitation loss bin per climate change scenario with removal of plant strategies**: (A) Precipitation loss 250-400 (mm) with increasing CO_2, (B) Precipitation loss 250-400 (mm) with fixed CO_2, (C) Precipitation loss 400-850 (mm) with increasing CO_2, (D) Precipitation loss 400-850 (mm) with fixed CO_2, (E) Precipitation loss greater than 850 (mm) with increasing CO_2, (F) Precipitation loss greater than 850 (mm) with fixed CO_2. The data underlying this figure are provided in figshare (https://doi.org/10.6084/m9.figshare.26232395).

Supplementary Notes (aDGVM2 Model Description) for Langan et al. "Amazon forest resistance to drought is increased by diversity in hydraulic traits" * †

*This appendix is in part published as an appendix to 'Langan et al.[11] Climate-biomes, pedo-biomes or pyro-biomes: which world view explains the tropical forest-savanna boundary in South America?' in the *Journal of Biogeography* (reproduced and modified with permission) and in Langan.[12] The order of residual soil water content (rc) values in Tab. S7 has been changed to correct a mis-specification in.[11] Text, references, and an additional figure has also been added to address reviewer comments.

†Author contributions: aDGVM2 was conceived by S.H. and S.S. and the initial implementation was written by S.S.. Model description text written by S.S. and L.L., text based on submodels shared between aDGVM1 and aDGVM2 is identical to that published in the aDGVM1 model description.[13] L.L. and S.H. redesigned plant hydraulics submodels and L.L. implemented the designed hydraulics submodels. L.L. redesigned the soil hydrology submodel. L.L. conceived the reproductive isolation functionality which was coded by S.S.. S.H. and L.L. redesigned and implemented grass architecture submodules. L.L. and S.S. designed the semi-spatial light competition functionality which was implemented by S.S.. S.H. and L.L. redesigned carbon allocation and leaf phenology submodules. L.L. modified plant recruitment and plant mortality submodels.

S1 Introduction and modelling concepts

This document provides a description of aDGVM2 (adaptive dynamic global vegetation model, version 2).[14] detailed the motivation for the development of this model as well as the novel features of aDGVM2. The following paragraphs summarize important features of aDGVM2. DGVMs commonly define vegetation using a limited number of plant function types (PFTs) and use point estimates for plant trait values[14,15] (Fig. S14). Collapsing observed variability in plant traits in this way may limit the potential of these models to explore past, present and future vegetation dynamics. Further, the way in which competition is represented in many DGVMs has been criticised[14,16] as competition is simulated at the PFT level rather than at the individual level where competitive processes operate.[17] aDGVM2 is an individual-based dynamic vegetation model where the growth, reproduction and mortality of individual plants is simulated. A novel feature of aDGVM2 is that each plant can have a specific and potentially unique combination of trait values that influence how a plant performs under given biotic and abiotic conditions. This implementation allows that the level where competitive and selective processes operate within our modelling framework moves from the level of the PFT or plant cohort to the level of the individual and trait.

The community of plants and their trait values evolve through time, this evolution is constrained by trade-offs between traits. Poorly performing individuals are continually removed from the plant population by mortality which results in a filtering of trait values. Further, the community and species' trait values can evolve through successive generations via reproduction, mutation and crossover which we approximate by using a genetic optimisation algorithm (GOA, Eq. S75). Additionally, we simulate reproductive isolation in the model by assigning individuals a 'species label' and restricting reproduction to individuals with the same species label. Previous simulations showed that reproductive isolation increases the likelihood that

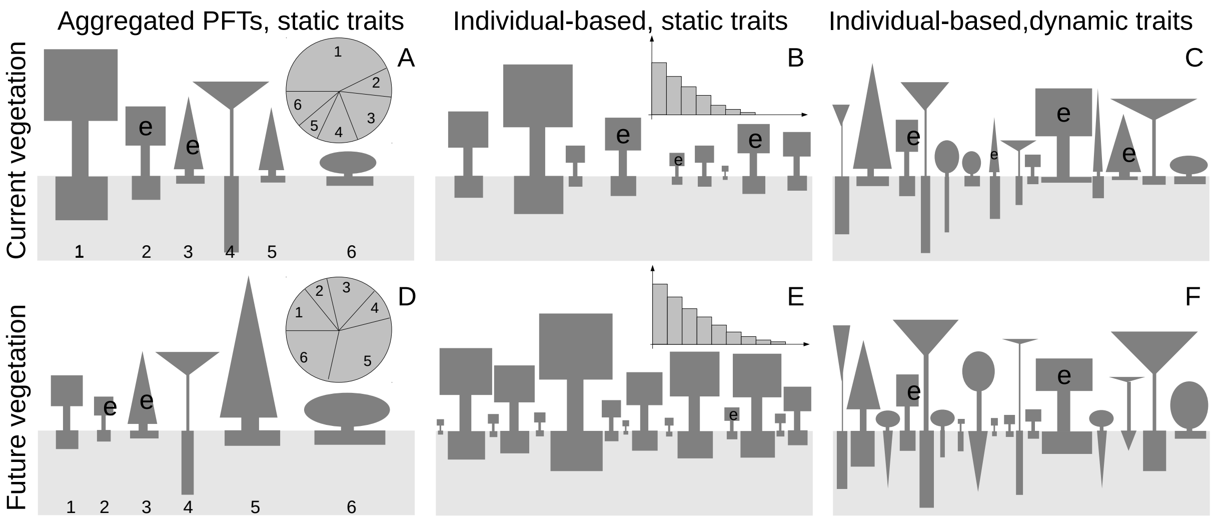

Figure S14: Conceptual model of representation of vegetation in DGVMs. (A) The model simulates six individuals where each individual represents a functional type. The number of individuals and their parameters are fixed. The dominant vegetation is defined by the relative abundance of the functional types. (B) The model is individual-based and each individual can differ in size or biomass while all individuals of one functional type have a similar trait combination. The dominant vegetation is defined by biomass and density of different functional types. (C) The model is individual-based and each individual has a unique combination of traits. Individuals can differ both in size or biomass and in trait values. The dominant vegetation is defined by the relative abundance of individuals. Panels (D), (E) and (F) depict how climate change influences vegetation. (D) The relative abundances of the functional types change. (E) The number of trees, biomasses and the height structure are modified. (F) Tree number, tree community and traits of individuals can be modified. The 'e' indicates an evergreen functional type.

multiple coexisting plant strategies emerge from simulations.[14] Thus, a plant community consisting of individuals and species with potentially novel and diverse trait values is assembled iteratively through time.

Modelling how plants respond to drought has proven to be a major challenge for earth system models and improving the representation of plant hydraulics may allow us to better predict how a changing climate will influence vegetation.[18] To meet this challenge we implemented a simplified version of the cohesion tension theory adopted by[19] where elements determining plant conductances are considered in series and implement a set of trait trade-offs which in-

fluence a plant's hydraulic strategy whereby hydraulic safety trades-off against xylem and leaf conductivity.[20]

One of the main aims of vegetation models is to investigate how climate and vegetation interact and define the historical, current and potential future distributions of vegetation. A second is to provide a dynamic representation of the land-surface as a component of earth system models.[14] By moving away from the paradigm of fixed-trait PFTs and improving the way we represent plant competition, hydraulics, community assembly, trait diversity and processes associated with co-existence we aim to iterate towards a more holistic representation of vegetation dynamics, improve our understanding of how biotic and abiotic interactions shape vegetation patterns and better elucidate how current patterns of vegetation will respond to climate change.

S2 Input data

To simulate vegetation we use site specific soil and climate data as input data. Topsoil texture data were obtained from a global 5×5 minute data set of selected soil characteristics.[21] Saturated water content (θ_{swc}), saturated hydraulic conductivity (K_{sat}), saturation suction (ϕ_{soil}), a parameter which influences the rate with which conductance declines as soil dries (r), and the residual content of the soil (rc), were then classified based on these soil texture data (Table S7). As in,[13] climate data were obtained from[1]'s global 10×10 minute data set of mean monthly surface climate data. We used precipitation (given by the mean value r_m and the coefficient of variance r_{cv}), wet-day frequency w_f, days with frost d_f, mean temperature $\bar{T}$, diurnal temperature range T_{Δ}, relative humidity h_s, sunshine percentage p_s, wind speed u_{ref} and elevation Z from this data set (Table S8).

These input data are used to calculate the secondary atmospheric characteristics, radiation,

photosynthesis and evapotranspiration of study sites. We follow[22]'s guidelines to calculate atmospheric pressure P, minimum and maximum temperature T_{min} and T_{max}, day temperature T, average saturation vapor pressure e^A, saturation vapor pressure e^S, slope of the vapor pressure curve s, vapor pressure deficit h_{vpd}, psychrometric constant γ, density of air ρ_{air}, photosynthetic active radiation Q_p and the net radiation Q_0 (Table S8). The rainfall algorithm[1] generates a time series of daily rainfall F_i for each year from the parameters r_m and r_{cv}. Characteristics of the input data from the database and other variables characterising the environment are summarized in Table S8.

S3 Model description

S3.1 Leaf physiology

The following sections describe how we estimate the daily leaf-level photosynthetic and respiration rates of study sites from temperature, relative humidity, atmospheric pressure, wind speed and photosynthetically active radiation. We link sub-models for photosynthesis and for stomatal conductance.

S3.1.1 Photosynthesis sub-model

We follow the Collatz[23,24] implementation of the Farquhar[25] model of leaf photosynthesis to calculate the (bio-physical) gross and net photosynthetic rates A_0^b and A_n^b (units μmol m^{-2}s^{-1}). Maximum light saturated rate of photosynthesis A_{max} is fixed to 30 μmol m^{-2}s^{-1}.[26] The maximum light saturated rate of photosynthesis A_{max} is used to estimate the maximum carboxyla-

tion rate V_{max} (μmol m^{-2}s^{-1}) as

$$V_{max} = 2^{0.1(T-25)} A_{max} \cdot A_S \frac{1}{\left(1 + e^{0.3(13-T)}\right)\left(1 + e^{0.3(T-36)}\right)}, \tag{S1}$$

where T is the (leaf) temperature[24] and A_S is a global scaling factor for both C_3 and C_4 photosynthesis.[24]

For calculating the biophysical rate of photosynthesis we prescribe the internal CO_2 partial pressure, c_i, using simplifying assumptions. For C_3 plants, c_i is taken to be 70% of atmospheric partial pressure.[27] For C_4 plants, c_i represents the bundle sheath value. There is no consensus on how to chose c_i for C_4 plants and we estimated c_i to be eight times the atmospheric partial pressure of CO_2 even though in our simulations, C_4 photosynthesis is not sensitive to the bundle sheath value as photosynthesis is not CO_2 limited, see section (S3.1.3) for how c_i is estimated by linking it to a diffusion gradient model of photosynthesis. The CO_2 compensation point is defined as

$$\Gamma_* = \frac{O_i}{2\tau}, \tag{S2}$$

where τ describes the partitioning of RuBP to the carboxylase or oxygenase reactions of Rubisco and O_i is the inter-cellular partial pressure of oxygen (assumed to be 21 kPa). Further, K_c is the Michaelis constant for CO_2 and K_o is the O_2 inhibition constant. We use the function

$$f_{25}(T) = K_{25} \cdot Q_{10}^{\frac{T-25}{10}}, \tag{S3}$$

to describe the response of K_c, K_o and τ to temperature T. Here, K_{25} and Q_{10} are empirically determined parameters specific for K_c, K_o and τ (see Table S11).

The gross rate of photosynthesis A_0 is calculated, following[23] for C_3 plants and[24] for C_4 plants, as the minimum of three potentially limiting assimilation rates. The Rubisco limited assimila-

tion rate J_c is defined as

$$J_c = \frac{V_{max} \cdot (c_i - \Gamma_*)}{c_i + K_c(1 + O_i/K_o)}, \tag{S4}$$

$$J_c = V_{max}, \tag{S5}$$

for C_3 and C_4 respectively. When light is limiting, the efficiency of CO_2 fixation is limited by the quantum yield. The light limited assimilation rate J_e is defined as

$$J_e = a \cdot \alpha \cdot Q_0 \cdot \left(\frac{c_i - \Gamma_*}{c_i + 2\Gamma_*} \right), \tag{S6}$$

$$J_e = a \cdot \alpha \cdot Q_0, \tag{S7}$$

for C_3 and C_4 respectively. Here Q_0 is the incident quantum flux density (μmol m^{-2}s^{-1}) that a leaf receives, a is the leaf absorptance and α is the intrinsic quantum yield of photosynthesis (see Table S11). When light and Rubisco do not limit the assimilation rate, then it is assumed that the capacity for the export of the products of photosynthesis is limiting for C_3 plants. This transport limited assimilation rate J_s is approximated as

$$J_s = \frac{V_{max}}{2}. \tag{S8}$$

For C_4 plants, when light and Rubisco are not limiting it is assumed that CO_2 concentrations limit the assimilation rate. This CO_2 limited rate J_p is approximated as

$$J_p = \frac{\kappa \cdot c_i}{P}, \tag{S9}$$

.[28] The term κ is the empirically defined initial slope of the response of CO_2 to photosynthesis

(units μmol m^{-2} s^{-1}) and P is the atmospheric pressure (Pa). In summary, the gross rate of (bio-physical) photosynthesis A_0^b is

$$A_0^b = \min(J_c, J_e, J_s), \quad \text{(S10)}$$

$$A_0^b = \min(J_c, J_e, J_p), \quad \text{(S11)}$$

for C_3 plants and for C_4 plants. The net rate of (bio-physical) photosynthesis A_n^b is

$$A_n^b = A_0^b - R_{mLs}, \quad \text{(S12)}$$

where

$$R_{mLs} = r \cdot V_{max}, \quad \text{(S13)}$$

is the single leaf maintenance respiration rate. Here, r is a proportion assumed to be 0.015 for C_3-photosynthesis and 0.025 for C_4-photosynthesis[23,24] and V_{max} is the maximum carboxylation rate from Equation (S1). All parameters and variables for section (S3.1.1) are summarized in Table S11.

S3.1.2 Stomatal conductance sub-model

The CO_2 assimilation rate is coupled to stomatal conductance using Ball's[29] empirical model. The model relates the response of stomatal conductance g_s (μmol m^{-2}s^{-1}) to the net rate of CO_2 uptake A_n:

$$g_s = m\frac{A_n \cdot h_s \cdot P}{c_s} + b. \quad \text{(S14)}$$

The terms m and b are empirically derived parameters (see Table S12), h_s is the relative humidity (expressed as unitless ratio), P is the atmospheric pressure (Pa) and c_s is the partial

pressure of CO_2 at the leaf surface, calculated as

$$c_s = c_a - \frac{1.4 \cdot A_n \cdot P}{g_b}. \tag{S15}$$

Here, c_a is the atmospheric partial pressure of CO_2 (Pa) and g_b is the leaf boundary layer conductance, estimated as

$$g_b = 0.271 \cdot 10^6 \sqrt{\frac{u(z)}{D_L}}, \tag{S16}$$

where, $u(z)$ is the wind speed (ms^{-1}) at height z (m) above the ground and D_L is the characteristic leaf dimension.[30] We calculate the wind speed $u(z)$ from the reference wind speed u_{ref} (m s^{-1}), measured at height z_{ref} (m) above the ground as

$$u(z) = u_{ref} \frac{\ln(z - z_d) - \ln(z_0)}{\ln(z_{ref} - z_d) - \ln(z_0)}. \tag{S17}$$

Here, z_d is the displacement height (m) and z_0 is the roughness length (m).[30] Both z_0 and z_d are functions of the aerodynamic properties of the vegetation and following Jones[30] we simply assume $z_d = 0.86 \cdot \bar{H}$ and $z_0 = 0.06 \cdot \bar{H}$. For these purposes we assume mean vegetation height $\bar{H}$ is 1.5m. The reference height z_{ref} is 10 m and the wind speed u_{ref} is read from a database. Variables in section (S3.1.2) are summarized in Table S12.

S3.1.3 Linking photosynthesis and stomatal conductance

The leaf photosynthesis and conductance sub-models are interdependent. The photosynthesis model requires estimates of c_i, which is determined by stomatal conductance. The stomatal model, in turn requires estimates of A_n, which also depends on c_i. The system of equations is

closed by noting that A_n can also be defined in terms of the CO_2 diffusion gradient

$$A_n^d = \frac{g_s \cdot (c_s - c_i)}{1.6 \cdot P}. \tag{S18}$$

When solving for A_n we iteratively seek the value of c_i that satisfies both Equation (S18) and the equations

$$A_n^b = \min(J_c, J_e, J_s) - R_{mLs}, \tag{S19}$$

$$A_n^b = \min(J_c, J_e, J_p) - R_{mLs}, \tag{S20}$$

for C_3 and C_4 plants given by Equations (S10) or (S11) and (S12), hence, we solve the equation

$$c_i^* = \min_{c_i > 0} \left| A_n^b - A_n^d \right|. \tag{S21}$$

S3.2 Single plant biomass pools

aDGVM2 is individual-based and simulates the dynamics of trees and grasses. Each plant consists of different compartments; we simulate biomass pools for leaves B_l, stem B_s, bark B_b, root B_r, reproduction B_p and storage B_t. Leaf biomass is required for photosynthetic carbon gain, stem biomass creates the structure required to capture light efficiently, bark biomass protects vegetation against fire, root biomass is required for water uptake, reproduction biomass is used for seed production and storage biomass is required for leaf flush after dormancy and for re-sprouting after fire. For grasses, we simply assume that $B_s = B_b = 0$. More details of the functions of the different compartments are provided in the following sections. We further simulate dead leaf biomass which accumulates when plants move from the metabolic to the dormant state. The dead leaf biomass pool is required to calculate the fire intensity (see

section S3.13).

S3.3 Tree architecture

S3.3.1 Canopy architecture

Plant height H is calculated as

$$H = \mathrm{e}^{\frac{\log(B_s)+b_1}{b_2}}, \tag{S22}$$

where B_s is the stem biomass. The parameters b_1 and b_2 describe the relation between aboveground biomass and plant height and they are subject to change as defined by the GOA (Table S9 provides trait value ranges). Plant height and aboveground biomass are used to calculate the stem diameter. We assume that the stem is a cylinder with homogeneous wood density ρ_{wood}. Wood density is calculated as a function of P_{50}, the matric potential at which a 50% loss of xylem conductivity occurs (see section S3.6.1).

The inner stem diameter, that is the stem diameter excluding bark is given as

$$D_i = 2\sqrt{\frac{B_s}{\pi \cdot \rho_{wood} \cdot H}}, \tag{S23}$$

the overall stem diameter including the bark is given as

$$D_s = 2 \cdot \sqrt{\frac{B_s + 2 \cdot B_b}{\pi \cdot \rho_{wood} \cdot H}}. \tag{S24}$$

Here we simply assume that the bark density is $\rho_{wood}/2$. The difference between the diameters D_i and D_s defines the bark thickness as $0.5 \cdot (D_s - D_i)$ which influences the fire sensitivity of plants (see S3.13).

Based on the equation used by,[31] tree stem diameter is used to calculate the canopy radius r_c at

height z above the ground by using the equation

$$r_c(z) = C_1 \cdot D_i \left(1 - \left(\frac{z}{H}\right)^{C_2}\right). \tag{S25}$$

Here, C_1 is the ratio between the canopy radius at the ground ($z = 0$) and stem diameter and C_2 defines the shape of the canopy, thus, $C_2 = 1$ defines a cone shaped canopy, $C_2 > 1$ defines a more cylinder shaped canopy and $C_2 < 1$ defines a copped canopy. The parameter C_1 is fixed at 10.0 and parameter C_2 is a trait defined by the GOA (Table S9). Canopy area at the ground, $D_c(0)$, has a minimum value of 0.2m^2. Canopy area at height z above the ground is then given by

$$D_c(z) = r_c(z)^2 \cdot \pi. \tag{S26}$$

where tree leaf area index L_{tree} (LAI) is calculated as

$$L_{tree} = \frac{B_l \cdot A_{SL}}{D_c(0)}. \tag{S27}$$

Here, B_l is the leaf biomass, $D_c(0)$ is the canopy area at the ground and A_{SL} is the specific leaf area (SLA).

S3.3.2 Stem architecture

Equations (S22) and (S24) describe how plant height and stem diameter are obtained from biomass. These equations use three traits to describe plant allometry, b_1, b_2 and ρ_{wood}. These parameters describe whether plants grow preferentially in height which implies low stem diameters or whether they grow preferentially in diameter which implies low plant height. These differences in stem architecture imply a trade-off between water transport capacity, light

availability and mechanic stability. Thus, a tall stem with small diameter implies high light availability and an advantage in light competition, however, it is mechanically unstable and does potentially not allow to transport the amounts of water required by the plant. In contrast, plants with high stem diameters are mechanically stable and not susceptible breakage however, they are generally smaller and potentially shaded by other plants.

To be mechanically stable, plant height H, stem diameter D_s and wood density ρ_{wood} must fulfil a stability condition. Following Niklas[32] we calculate the critical buckling height (in m) as

$$H_{crit} = 0.79 \left(\frac{11.852 \cdot \rho + 37}{9.81} \cdot \rho_{wood} \right)^{\frac{1}{3}} D_s^{\frac{2}{3}}. \tag{S28}$$

When the plant height exceeds the critical height H_{crit}, then the mortality probability of the plant increases (see section S3.12). Section (S3.6.1) describes the hydraulic trade-offs associated with tree architecture.

S3.4 Grass architecture

Grass architecture in DGVMs has received little attention. Here we implement a novel grass architecture scheme where leaf width L_w (mm) is calculated using Craine et al.'s[33] constraint line and is a function of P_{50}, the matric potential where 50% loss of xylem conductivity occurs,

$$L_w = 1.25 \cdot P_{50} + 22.5. \tag{S29}$$

We then use the relationship between leaf tissue density L_{TD} and P_{50} in Tucker et al.[34] to calculate L_{TD} (in g mm^{-3})

$$L_{TD} = (-0.025 \cdot P_{50} + 0.294) \cdot 0.001. \tag{S30}$$

Leaf volume L_v (mm^3) is calculated as

$$L_v = \frac{0.7 \cdot 1000 \cdot B_l}{L_{TD}}. \tag{S31}$$

We assume a leaf thickness L_t of 0.1 (mm) and calculate total leaf length L_{LT} (m) as

$$L_{LT} = \frac{L_v}{L_t \cdot L_w}. \tag{S32}$$

Grass leaf area $L_{A^{grass}}$ (m^2) is

$$L_{A^{grass}} = \frac{L_w}{1000} \cdot L_{LT}. \tag{S33}$$

Grass specific leaf area $A_{SL_{grass}}$ (m^2 kg^{-1}) is calculated by setting B_l to 1 kg. Canopy diameter D_{grass} (m) is assumed to be a function of root biomass

$$D_{grass} = 0.5 + 1.5 \cdot \frac{B_r}{B_r + 0.250}. \tag{S34}$$

Grass canopy area D_c (m^2) is assumed to have a maximum of 1m^2 and is calculated as

$$D_c = \min\left(1, \pi \cdot \left(\frac{D_{grass}}{2}\right)^2\right), \tag{S35}$$

making grass leaf area index L_{grass} (LAI)

$$L_{grass} = \frac{L_{A^{grass}}}{D_c}. \tag{S36}$$

S3.5 Light availability and light competition

Light availability of an unshaded plant is described using Beer's law, that is

$$Q_0 = \int_0^L e^{-\omega L} \, dL = \frac{1}{\omega} \cdot \left(1 - e^{-\omega L}\right), \tag{S37}$$

where L is an individual's leaf area index and the parameter ω describes the light extinction in the canopy, we assume higher density leaves result in higher light extinction and calculate ω as a function of specific leaf area

$$\omega = 0.3 + \frac{A_{SL^{max}}}{A_{SL^{max}} \cdot A_{SL}}. \tag{S38}$$

where the maximum permissible SLA ($A_{SL^{max}}$) is 32.67 (m^2kg^{-1}) which corresponds to the maximum value of P_{50} allowed, see section (S3.6.1).

To simulate light competition, we assume that plants are arranged on an equidistant rectangular grid. The size of a grid-cell is dependant on the plot area and the number of individuals we simulate. Light availability of a target plant is influenced by eight neighbours. When the canopy height of the target plant H_t^c is less then the canopy height of a neighbour plant, H_n^c, the neighbour has a canopy area greater than the size of a grid-cell on our equidistant grid, here 2.77m^2, and the neighbour has an LAI greater than 0.1 then light availability of the target plant is reduced and calculated as

$$Q_t = Q_0 \cdot \mu \cdot \left(1 - \frac{H_t^c}{H_n^c}\right)^{L \cdot min\left(1, \frac{D_c(0)}{25}\right)}. \tag{S39}$$

Here, $\mu = 0.5$, describes the maximum impact of a neighbour plant on the light environment of the target plant, L is the leaf area index of a neighbour plant and 25 is the point at which a neighbours canopy area at the ground, $D_c(0)$, is greater than the summed area of it's own grid-

cell and the 8 surrounding grid-cells given the number of simulated individuals is 3600 and the plot size is 10,000m^2. Thus, the light availability of an individual is influenced by it's height relative to it's neighbours as well as the leaf area index and canopy area of it's neighbours. This procedure is repeated for the eight neighbours of a plant. The canopy height is defined as

$$H^c = H \cdot \sqrt[C_1]{0.5}, \tag{S40}$$

to account for different canopy forms. The canopy height of more cylinder shaped canopies is almost the plant height while the canopy height of more copped canopies is less than the plant height.

S3.6 Root architecture and water competition

In the model we assume that for both trees and grasses the distribution of roots in the soil is described by the function

$$R_F(z) = R_n \left(1 - \left(\frac{z}{R_m}\right)^{R_1}\right)^{R_2}, \tag{S41}$$

where $R_F(z)$ is the fraction of roots at depth z, R_m is the maximum rooting depth of the plant, R_1 and R_2 describe the root form and R_n is a scaling factor that ensures that

$$\int_0^{R_m} R_F(z)\, dz = 1. \tag{S42}$$

This function allows us to simulate various root forms.

The parameters R_1, R_2 and R_m are traits defined by the GOA (Table S9). In the model we do not directly use Equation (S41) to describe the root form. We rather assume that roots in different soil layers are represented by cylinders. The proportion of roots R_{Fi} in soil layer i

($i = 1, 2, \ldots n$, where n is the number of soil layers is calculated by using Equation (S41)) is,

$$R_{Fi} = R_F(D_i). \tag{S43}$$

Here, D_i is in the average depth of soil layer i. The actual root fractions in soil layer i is then defined as

$$R^*_{Fi} = R_n \cdot R_{Fi} \cdot T_i, \tag{S44}$$

where T_i is the thickness of the soil layer i and R_n is a scaling factor that ensures that

$$\sum_{i=1}^{n} R^*_{Fi} = 1. \tag{S45}$$

S3.6.1 Plant Hydraulic Strategies - safety versus efficiency trade-offs

The plant water availability sub-models are crucial for modelling vegetation dynamics as well as predicting how vegetation formations may change in response to climate change. Vegetation models typically calculate plant water availability as a function of relative soil moisture content and root fractions across a number of soil layers, typically to a depth not greater than 3m.[36] In many areas of the world, observed plant rooting depths exceed those used in vegetation models; this discrepancy between observed and modelled rooting depths can have implications for plant water availability.[36] Experiments where precipitation has been artificiality reduced to examine the response of tropical forests to drought have demonstrated dramatic reductions in tree biomass with the highest mortality rates exhibited by large trees.[37,38] Worryingly, a number vegetation models appear to under represent the magnitude of these observed responses to drought.[39,40] However, progress at understanding drought induced vegetation responses in vegetation models is improving rapidly.[41] The accurate representation of plant water relations requires sub-models of plant hydraulics.

Modelling plant water transport though the soil-plant-atmosphere hydraulic continuum requires consideration of multiple plant compartments.[42] Implementations of the cohesion-tension theory of plant water ascent using an Ohm's law analogy have been shown to mimic important plant hydrological phenomena.[19,43] We implement a simplified version of the cohesion tension theory adopted by[19] where elements determining plant conductances are considered in series. In order to properly constrain emergent plant hydraulic strategies in aDGVM2 we consider the conductances of the following hydraulic elements; soil-root, root, sapwood and leaf. We implement a set of trait trade-offs which define a plant's hydraulic strategy in which hydraulic safety trades-off against xylem and leaf conductivity.[20] We detail this implementation below.

The matric potential at which a 50% loss of xylem conductivity occurs, P_{50}, is a key trait regulating plant hydraulic strategies. In aDGVM2, P_{50}, is a plant trait defined by the GOA (Table S9) with values ranging from -3.0 to -0.2 MPa. We use the empirical relationships described in[20] to describe relationships between P_{50}, wood density (ρ_{wood}), leaf dry matter content (L_{DMC}), sapwood conductivity (k_{sw}) and leaf conductivity (k_{leaf}). We calculate ρ_{wood} as

$$\rho_{wood} = 0.259 + (-0.05921 \cdot P_{50}) \cdot 10^3 \cdot 1.55, \quad \text{(S46)}$$

where 10^3 and 1.55 convert to ρ_{wood} to kg m^{-3}. L_{DMC} (g g^{-1}) is calculated as

$$L_{DMC} = 0.224 + (-0.041 \cdot P_{50}). \quad \text{(S47)}$$

L_{DMC} in turn defines tree specific leaf area $A_{SL_{tree}}$ (m^2 kg^{-1})

$$A_{SL_{tree}} = \frac{1}{L_{DMC} \cdot L_T}, \quad \text{(S48)}$$

L_T (mm) is tree leaf thickness, we assumed tree leaf thickness increases as a function of P_{50} where,

$$L_T = \frac{0.0224 - (0.041 \cdot P_{50})}{0.224 - (0.041 \cdot P_{50})}. \tag{S49}$$

Sapwood conductivity k_{sw} (kg s^{-1} m^{-1} MPa^{-1}) is calculated as

$$k_{sw} = \frac{581.85 + (90.07 \cdot P_{50} \cdot 18)}{10^3} \cdot \frac{A_{sw}}{H}, \tag{S50}$$

where H (m) is plant height and sapwood area A_{sw} (m^2) is calculated following[44] as

$$A_{sw} = \frac{1.582 \cdot D_s^{1.764}}{10^4}, \tag{S51}$$

where D_s is stem diameter. Leaf conductivity k_{leaf} (kg s^{-1} m^{-1}) is calculated as

$$k_{leaf} = \frac{61.993 + (7.758 \cdot P_{50} \cdot 18 \cdot 0.001)}{10^3} \cdot A_{leaf}, \tag{S52}$$

where A_{leaf} is leaf area (m^2) and is calculated as

$$A_{leaf} = D_c(0) \cdot L. \tag{S53}$$

Here, $D_c(0)$ is the canopy area at the ground and L is leaf area index.

S3.6.2 Water transport

We calculate plant water availability, G_w, as follows

$$G_w = \min\left(1, \frac{E_{canopy} \cdot G}{E_t^p}\right), \tag{S54}$$

where E_t^p is a plant's evapotranspiration calculated using Eq. (S83) and E_{canopy} is the amount of water which can be extracted from the soil and transported through an individual for the purpose of transpiration,

$$E_{canopy} = \frac{\Delta\Psi}{(R_{sw} + R_{leaf} + R_{root} + R_{soil-root}) \cdot \eta}. \tag{S55}$$

The viscosity of water, η, is taken to be 1.00. R_{sw} and R_{leaf} are sapwood and leaf resistivity while R_{root_i} and $R_{soil-root_i}$ are the root and soil to root resistances of the ith soil layer. For grasses, R_{sw} is omitted. The potential difference driving transpiration, $\Delta\Psi$ (MPa), is calculated as

$$\Delta\Psi = P_{50} - \frac{H \cdot \rho_{water} \cdot g}{10^6} - \Psi_{soil}, \tag{S56}$$

here H is plant height, ρ_{water} is the density of water (999.97 kg m^3), g is acceleration due to gravity (9.8 m s^{-2}) and 10^{-6} scales to MPa. Ψ_{soil} is the average soil matric potential an individual experiences across it's root profile and is calculated as

$$\Psi_{soil} = \sum_{i=1}^{n} \Psi_{soil_i} \cdot R_{Fi}. \tag{S57}$$

The soil matric potential of the ith soil layer, Ψ_{soil_i}, is calculated following[45] as

$$\Psi_{soil_i} = \frac{-\phi_{soil} \cdot es^{-r}}{100}, \tag{S58}$$

where es is the effective saturation of the soil

$$es = \frac{\theta_i - rc}{\theta_{swc} - rc}, \tag{S59}$$

θ_i is the soil water content in soil layer i, rc is the residual content,[46] ${\theta_{swc}}$[47] is the saturated water content. Saturation suction, ϕ_{soil},[48] r,[49] rc and θ_{swc} are soil texture specific parameters, see Table S7.

We calculate R_{sw} as

$$R_{sw} = \frac{1}{k_{sw}}, \tag{S60}$$

and R_{leaf} as

$$R_{leaf} = \frac{1}{k_{leaf}}, \tag{S61}$$

where k_{sw} and k_{leaf} are sapwood and leaf conductivity as defined in Equations (S50) and (S52).

Root resistance across a plant's root profile R_{root} is calculated by summing the resistances across all soil layers and is calculated following[42]

$$R_{root} = \sum_{i=1}^{n} R_{root_i}, \tag{S62}$$

where

$$R_{root_i} = \frac{1}{k_{root_i}}, \tag{S63}$$

k_{root_i} (kg kg^{-1} s^{-1} MPa^{-1}) is calculated as a function of fine root biomass B_{Fr}. We assume that 20% and 90% of total root biomass is fine root biomass for trees and grasses respectively and we calculate conductance as

$$k_{root_i} = 0.0004 \cdot B_{Fr} \cdot R_{Fi}, \tag{S64}$$

where R_{Fi} is the proportion of an individual's root biomass in soil layer i. Soil to root resistance $R_{soil-root}$ (m^2 MPa s kg^{-1}) is calculated by summing the resistances across the all soil

layers and is calculated following[50]

$$R_{soil-root} = \sum_{i=1}^{n} R_{soil-root_i}, \tag{S65}$$

and

$$R_{soil-root_i} = \log\left(\frac{\sqrt{\frac{1}{L_i \cdot \pi}}/r}{2 \cdot \pi \cdot L_i \cdot K_{soil_i} \cdot g}\right), \tag{S66}$$

where L_i is the root length (m) in the ith soil layer, r is the root radius (m) which is set to 0.0005m.[51] K_{soil_i} is soil hydraulic conductivity of the ith soil layer and is calculated using Eq. (S82). Again following,[50] we calculate root length L_i for the ith soil layer as

$$L_i = \frac{B_{Fr} \cdot R_{Fi} \cdot 1000}{\rho_{root} \cdot \pi \cdot r^2}, \tag{S67}$$

where ρ_{root} is the density of root material (g m^{-3}). We assume root material density is the same as wood density ρ_{wood}.

In aDGVM2 it is assumed that xylem vulnerability to cavitation regulates transpiration by reducing canopy water supply, we thus scale plant water supply by a measure of xylem cavitation G,

$$G = 1 - \frac{1}{1 + \mathrm{e}^{a(\Psi_{soil} - P_{50_{root}})}}, \tag{S68}$$

where a is an empirically defined parameter which determines the slope of the G curve and is currently set to 5. $P_{50_{root}}$ defines the point at which 50% of conductance is lost

$$P_{50_{root}} = P_{50} + 0.2 + (H \cdot \rho_{water} \cdot g) \cdot 10^{-6}, \tag{S69}$$

where H is plant height, ρ_{water} is the density of water (999.97 kg m^3), g is acceleration due to gravity (9.8 m s^{-2}) and 10^{-6} scales to MPa. Roots usually cavitate at less negative matric

potentials than stems, we thus add 0.2 MPa to P_{50} to account for this.

S3.7 Carbon balance

Leaf level photosynthetic rates A_0 are scaled to the canopy level by using the plant's light and water availability. Light competition is described by a parameter Q_t (see section S3.5, water stress is described by a parameter G_w (see section S3.6). The canopy photosynthetic rate is then given by

$$A = Q_t \cdot G_w \cdot D_c(0) \cdot A_0. \tag{S70}$$

Respiration is simulated as in aDGVM[13] and we do not provide all details here. The respiration model separates between growth and maintenance respiration. Growth respiration is a fixed fraction of the photosynthetic carbon gain, maintenance respiration is defined by biomass, C:N ratios and temperature and it is calculated separately for each biomass pool. In the model it is assumed that bark has the maintenance costs as stem biomass, reproduction biomass has the maintenance costs as leaf biomass and storage has the maintenance costs as root biomass. When plants are in the metabolic state, then the net carbon balance is given by

$$\Delta C = A - R_g - R_m, \tag{S71}$$

where A is the water and light limited canopy photosynthetic rate, R_g is the growth respiration and R_m is maintenance respiration. When plants are in the dormant state, they do not have a photosynthetic carbon gain and they are only affected by maintenance respiration and turnover.

Turnover, that is the permanent mortality of leaf biomass, is a function of SLA and leaf biomass.

Each day, leaf biomass is reduced by a value

$$B_{turnover} = \frac{1}{29664 \cdot (A_{SL} \cdot 10)^{-0.909}} \cdot B_l. \tag{S72}$$

This function creates a tradeoff; leaves with high SLA ensure high photosynthetic rates while longevity is short. In contrast, leaves with low SLA have lower photosynthetic rates but higher longevity (Figure S17). Hence, the total photosynthesis of a leaf over it's lifespan can be similar for leaves with high and low SLA.

S3.8 Carbon allocation

The carbon gained by photosynthesis ΔC needs to be allocated to the different biomass pools of the plant (leaf, stem, bark, roots, storage, reproduction, Figure S18). Although there are complex models that describe carbon allocation based on the environmental conditions and deficits of the plants,[52,53] we use a simple carbon allocation scheme in aDGVM2. We assume that carbon allocation to different biomass pools is given by fixed parameters A_p, where p indicates one of the biomass pools. All A_p must sum to one. The parameters A_p differ between single plants and they are adjusted by the GOA (Table S9). This allocation rule implies a mass balance trade-off as an increase of allocation to one pool implies a decrease of allocation to at least one other pool. Allocation to leaf is assumed to be dynamic and a function the plant's leaf area index

$$A_{leaf}^{dyn} = \frac{A_{leaf}}{1 + \mathrm{e}^{c_1(L-c_2)}}, \tag{S73}$$

where c_1 and c_2 are constants (c_1=3, c_2=7). This dynamic allocation function ensures that carbon allocation to leaves and leaf growth is high when plants move from the dormant to the metabolic state (see section *"Leaf phenology"*) or after fire has removed leaf biomass and that allocation to leaves decreases when the leaf area index approaches seven. For higher LAI, ad-

ditional leaf biomass does not increase carbon gain substantially because Q_0 (Eq. S37) saturates. For trees we assume that as the difference between A_{leaf} and A^{dyn}_{leaf} is added to carbon allocation to stem A_s while for grasses this difference is distributed evenly across all plant compartments. We further assume that the individual's storage biomass pool size can not be larger than the root biomass pool and storage for trees and grasses cannot exceed 0.5 and 50 kg respectively, hence when $B_{storage} > B_{root}$ then $A_{storage} = 0$. This is to prevent excessively large storage pools from accumulating and allow dynamic allocation to the other carbon pools.

S3.9 Leaf phenology

Four phenology strategies are considered. Deciduous (soil matric potential triggered leaf flush and abscission), deciduous (light triggered leaf flush and abscission), evergreen (soil matric potential triggered leaf flush) and evergreen (light triggered leaf flush). Whether a plant is evergreen or deciduous is described by a trait P_E which is defined by the GOA (Table S9). Whether a plant's leaf flush is governed by soil matric potential or light is described by a trait P_S which is modified by the GOA (Table S9). Across the Amazon, it has been observed that many trees flush their leaves during the dry season when incident photosynthetically radiation increases.[54] We therefore include traits to capture this widespread phenomenon across the study area (T_{lu}, T_{ld}, Table S9).

A cost of cavitation is implemented whereby, if Ψ_{soil} (integrated over the plant's rooting profile, Equation S57) is more or less negative than P_{50} a cavitation counter C_{cav} is incremented or decreased. If this counter exceeds 30 then an unscheduled abscission event occurs. During an unscheduled abscission event 2% of leaf and root biomass is lost, this loss of biomass occurs daily until either biomass approaches zero or C_{cav} is less than 30.

Deciduous trees abscise leaves according to the light or Ψ_{soil} triggers. During such a scheduled abscission we assume that a constant proportion (we assume 30%) of leaf biomass is

transferred to storage and the remainder is lost to leaf litter. For both evergreen and deciduous strategies, when leaf flush occurs (in accordance with the light or Ψ_{soil} triggers) carbon in the storage pool is used. When plants flush their leaves, a fraction A_{TL} of the storage biomass is allocated to leaf biomass to enable photosynthesis. The parameter A_{TL} is a trait and defined by the GOA (Table S9). The carbon cost of this reallocation is assumed to be 35%, i.e. the growth respiration cost.

For soil moisture triggered plants, when soil matric potential falls below a threshold T_{rd} for 7 days, then the plant moves into the dormant state. For light triggered plants, when light availability falls below a threshold T_{ld} for 7 days the plant moves into the dormant state. For deciduous strategies this leads to leaf abscission while for evergreen strategies it sets a phenology trigger to the off state. When soil matric potential across the rooting zone exceeds T_{rd} for 7 days or when light availability exceeds T_{lu} for 7 days, then leaf flush from storage is initiated and deciduous plants move from the dormant to the metabolic state. The parameter T_{rd} is constrained to be less negative than an individual's P_{50}. The parameters T_{rd}, T_{ld} and T_{ru} are traits defined by the GOA (Table S9).

S3.10 Reproduction, inheritance, mutation, and cross-over

One major feature of aDGVM2 is that each plant is characterised by an individual combination of trait values (Table S9). This trait combination is passed to the offspring of the plant. Additionally, trait combinations can be modified by mutation and cross-over. The model iteratively generates various trait combinations but only a limited set of these trait combinations can survive and reproduce under the given environmental conditions. Hence, the model iteratively assembles a set of trait combinations (or a plant community) that is optimally adapted to the environment (Fig. S19). The following paragraphs describe these processes in more detail.

The number of seeds produced by each plant ϕ is defined by the biomass in the reproduction pool B_P and by the seed weight W,

$$\phi = \left\lfloor \frac{B_P}{W} \right\rfloor, \tag{S74}$$

where $\lfloor x \rfloor$ describes the integer value of x. The biomass ϕW is removed from the reproduction biomass pool B_P. The seed bank collects the seeds of all plants. Seeds store the trait combination of the parent plant. For reasons of model performance, we assume that a plant can add a maximum of 200 seeds into the seed bank; our seed bank, of size 720,000 can then store up to 3600 seeds with different trait combinations for trees and grasses respectively.

In the model we allow mutation and cross-over to modify the trait values of the seeds. We assume that $\mathbf{T}_i = (T_{i1}, T_{i2}, \ldots, T_{in})$ is the trait combination of a seed i, that is a vector with the n trait values. Here, the vector $\mathbf{T}_i$ only contains traits which are subjected to the modification; these are the traits given in Table S9.

S3.10.1 Mutation

Mutation means that trait values can randomly fluctuate. Mutation is defined by two parameters, (1) the mutation probability m_p and (2) the mutation rate m_r. The mutation probability a is pre-defined model parameter set to 0.01. For each trait of each seed, a random number between 0 and 1 is calculated, when this random number between is less than m_p then mutation will occur. When a trait value of a seed mutates, then a random number m_j in the interval between $1 - m_r$ and $1 + m_r$ is drawn for each trait in $\mathbf{T}$ and the new trait value T_j^* is obtained by multiplying old trait values with m_j, that is, $T_j^* = m_j \cdot T_j$. The mutation rate m_r is a predefined model parameter set to 0.05

S3.10.2 Cross-over

The cross-over algorithm used in aDGVM2 is based on the genetic optimisation algorithm DEoptim[55] as implemented in R.[56,57] The basic concept of the cross-over algorithm is to recombine trait combinations $\mathbf{T}_i$ to obtain a new set of trait combinations $\mathbf{T}_i^*$. Cross-over is restricted to seeds possessing the same species label. New trait combinations $\mathbf{T}_i^*$ are generated by using the original trait values $\mathbf{T}_i$ and values from a randomly drawn seed $\mathbf{T}_{random}$ which has the same species index. For each seed and each trait, crossover happens when a random number between 0 and 1 is less than the crossover probability C_p which is a fixed parameter set to 0.5. When cross-over occurs, then the new trait combination $\mathbf{T}_i^*$ is calculated as

$$\mathbf{T}_i^* = \mathbf{T}_i + c_f \cdot (\mathbf{T}_{random} - \mathbf{T}_i)\,, \tag{S75}$$

where c_f is a random number between 0 and 1 that is drawn for each trait of each seed. The cross-over routines in aDGVM2 ensure that trait values of a new seed are within the range of trait values of a plant's species.

S3.11 Plant recruitment

Once per year, new recruits are added to the plant population. As the plant population size N_{max} is fixed in the model, only a limited number of recruits can be added to the plant population. The probability of a grass or tree seed being drawn from the population of seeds is equal. The trait combination of a recruit is given by randomly selecting a seed from the seed bank. The availability of light to and weight of a seed can affect it's germination probability[58].[59] We thus calculate seed germination probability P_{germ} as

$$P_{germ} = (W + 0.04) - \left[0.15 \cdot \min\left(1, \frac{C_{gap}}{C_{eff}}\right)\right] \tag{S76}$$

where W is the seed weight and C_{gap} is the summed canopy area at the ground of neighbouring trees, see section S3.5, and C_{eff} is a constant set to 50 which affects the degree to which canopy closure alters germination probability.

S3.12 Plant mortality

Once per year, plant mortality is simulated. There are several potential reasons for plant mortality, (1) negative carbon balance, (2) insufficient height growth and (3) mechanic instability.

S3.12.1 Negative carbon balance

A plant is removed from the plant population when it has a negative carbon balance ΔC (Equation S71) and when a random number between zero and one is less than the mortality probability M_C.

S3.12.2 No height growth

A tree is removed from the plant population when it's height is below a threshold level of $M_B = 5$cm after 2 years. This rule removes trait combinations that cannot grow under the given environmental conditions.

S3.12.3 Mechanic instability

We assume that mechanic instability of tree stems can lead to buckling and plant mortality.[60] The probability for mortality as a result of mechanic instability P_i is defined as

$$P_i = \max\left(M_I\left(\frac{H}{H_{crit}} - 1\right)\right). \tag{S77}$$

where H_{crit} is the critical buckling height (eq. S28), H is the plant height and M_I is a mortality parameter, Table S10. When a random number between zero and one is less than P_i then a tree is removed from the plant population.

S3.13 Grass fire and tree topkill

The fire model used in aDGVM2 is similar to the fire model used in aDGVM.[13] By using fuel moisture θ_F, fuel biomass B_F and wind speed u_{ref} we calculate the potential fire intensity following[61] as

$$I(B_F, \theta_F) = hB_F \frac{\arctan(u_{ref}) \cdot c \cdot f(B_F, a_w)}{Q_m \cdot \theta_F + Q_v(1 - \theta_F)}. \tag{S78}$$

Here, Q_m and Q_v are heats of preignition of moisture and fuel, c is regression parameter and h is the heat yield of fuel consumed. Further,

$$f(B_F, a_w) = \frac{B_F}{B_F + a_w} \tag{S79}$$

is a sigmoidal function defined by a regression parameter a_w.

For a fire to occur on a particular day, the matric potential of the first soil layer must be less than -1.5 (MPa), precipitation must be less than 5 (mm), a random number between 0 and 1 must be less than P_{fire} where P_{fire} is calculated as

$$P_{fire} = 0.003 \cdot \max\left(0.01, \frac{\sum_{i=1}^{n} D_{c,i}(0)}{8000}\right). \tag{S80}$$

These conditions implicitly ensure that fuel is dry enough to carry fire. We assume that fire probability is higher in more open vegetation stands with low tree cover and that fire probability decreases as tree cover increases, here $D_{c,i}(0)$ is the canopy area at the ground of the ith tree.

Fire removes the total aboveground grass biomass, the total dead aboveground biomass and it can cause topkill, that is, damage of aboveground tree biomass. For fire to spread the fire intensity must exceed a minimum of 300 (kJ $s^{-1}m^{-1}$). Following[62] the probability of topkill is an empirically derived function of fire intensity I and tree height H,

$$P_{topkill}(H,I) = \frac{D_i}{D_s} \cdot \frac{\exp(D_1 - D_2 \cdot \ln(H) + D_3 \cdot \sqrt{I})}{1 + \exp(D_1 - D_2 \cdot \ln(H) + D_3 \cdot \sqrt{I})}, \tag{S81}$$

where D_i and D_s are the stem diameters excluding and including bark and D_1, D_2 and D_3 are regression parameters. By the multiplication with the stem diameters we simulate that bark protects vegetation against fire. This effect is supported by empirical evidence from Brazilian Cerrados,[61,63,64] yet it is weak in other savanna areas. Topkilled trees loose their aboveground biomass, however, storage biomass A_T allows them to re-sprout and to recover from the disturbance. To re-sprout, plants allocate fractions A_{TS} and A_{TL} of the storage biomass to stem and leaf biomass. The fractions A_{TS} and A_{TL} are traits and defined by the GOA.

S3.14 Water balance

To describe the soil water balance, we use a multi-layer tipping bucket model of soil moisture, similar to the model used in aDGVM.[13] In aDGVM2, rainfall is tipped from one layer into the next deeper layer when the soil moisture content exceeds the saturated water content θ_{swc}. Percolation between soil layers is calculated following Campbell (1974) using the equation

$$K_{actual_i} = K_{sat} \cdot \left(\frac{\theta_i}{\theta_{swc}}\right)^{2r+2} \tag{S82}$$

where K_{actual_i} is the non-saturated hydraulic conductivity of soil layer i, K_{sat} is saturated hydraulic conductivity, θ_i is the actual soil moisture content of soil layer i, θ_{swc} is the saturated

soil moisture content while r determines the rate at which percolation decreases as soil dries. The parameters K_{sat}, θ_{swc} and r are related to soil texture, Table S7. The amount of water percolating from one soil layer to the next is calculated daily.

A plant's evapotranspiration is calculated using the Penman-Monteith equation,[22,30]

$$E_t^p = \frac{s \cdot Q_0 + 86400 \cdot \rho_{air} \cdot c_p \cdot h_{vpd} \cdot g_b^c}{\lambda \left(s + \gamma \left(1 + \frac{g_b^c}{g_s^c} \right) \right)}. \quad \text{(S83)}$$

Here s is the slope of vapour pressure curve, Q_0 is the net radiation, ρ_{air} is the density of air, c_p is the specific heat of moist air, h_{vpd} is the saturation vapour pressure deficit, g_b^c is the canopy boundary layer conductance from eq. (S16), λ is the latent heat of air, γ is the psychrometric constant and g_s^c is the canopy stomatal conductance from eq. (S14). As in,[13] Allen et al.'s[22]'s detailed guide to the computation of the components of E_t^p is used.

Table S7: Soil properties. θ_{swc}: saturated water content; K_{sat}: saturated hydraulic conductivity; ϕ_{soil}: Saturation suction; r: shape parameter describing how percolation decreases as soil dries; rc: residual soil water content.

Soil Code HWSD	θ_{swc} (m^3 m^{-3})	K_{sat} (m hr^{-1})	ϕ_{soil} (m)	r	rc (m^3 m^{-3})
1	0.50	0.00133	0.405	12.13	0.090
2	0.52	0.0037	0.49	10.21	0.056
3	0.52	0.0037	0.49	10.21	0.090
4	0.51	0.0057	0.356	8.16	0.040
5	0.48	0.0043	0.63	8.16	0.075
6	0.48	0.022	0.63	3.63	0.015
7	0.48	0.0161	0.786	5.42	0.015
8	0.44	0.0014	0.153	9.39	0.109
9	0.46	0.0155	0.478	6.10	0.027
10	0.43	0.0113	0.356	6.77	0.068
11	0.45	0.0503	0.218	4.73	0.041
12	0.46	0.0967	0.09	4.32	0.035
13	0.46	0.1081	0.121	3.91	0.020
Nachtergaele et al.[21]	Saxton & Rawls[47]	Saxton & Rawls[47]	Clapp & Hornberger[48]	Boone et al.[49]	Rawls et al.[46]

Table S8: Input data from databases and secondary environmental variables. Secondary environmental variables are calculated as in.[13] "variable" indicates that this parameter is a modelled variable.

Name	Description	Value	Units
T_Δ	Daily temperature range	database	°C
$\bar{T}$	Mean day temperature	database	°C
w_f	Wet day frequency	database	frequency
r_m	Mean value of rain	database	$\mathrm{mm}/\mathrm{month}$
r_{cv}	Coefficient of variance of rain	database	%
p_s	Percentage of sunshine per day	database	%
h_s	Relative humidity	database	%
d_f	Frost days per month	database	$\mathrm{days}/\mathrm{month}$
u_{ref}	Reference wind speed	database	m/s
Z	Elevation	database	m
P	Atmospheric partial pressure	variable	Pa
T	Day temperature	variable	°C
T_{min}	Minimum temperature	variable	°C
T_{max}	Maximum temperature	variable	°C
s	Slope of vapor pressure curve	variable	kPa/C
γ	Psychrometric constant	variable	kPa/C
e^A	Actual vapor pressure	variable	kPa
e^S	Saturation vapor pressure	variable	kPa
ρ_{air}	Density of air	variable	g/m^3
h_{vpd}	Saturation vapor pressure deficit	variable	kPa
Q_p	Photosynthetically active radiation	variable	$\mu\mathrm{mol}/\mathrm{m}^2\mathrm{s}$
Q_0	Net radiation	variable	$\mu\mathrm{mol}/\mathrm{m}^2\mathrm{s}$
F_i	Simulated precipitation at day i	variable	mm

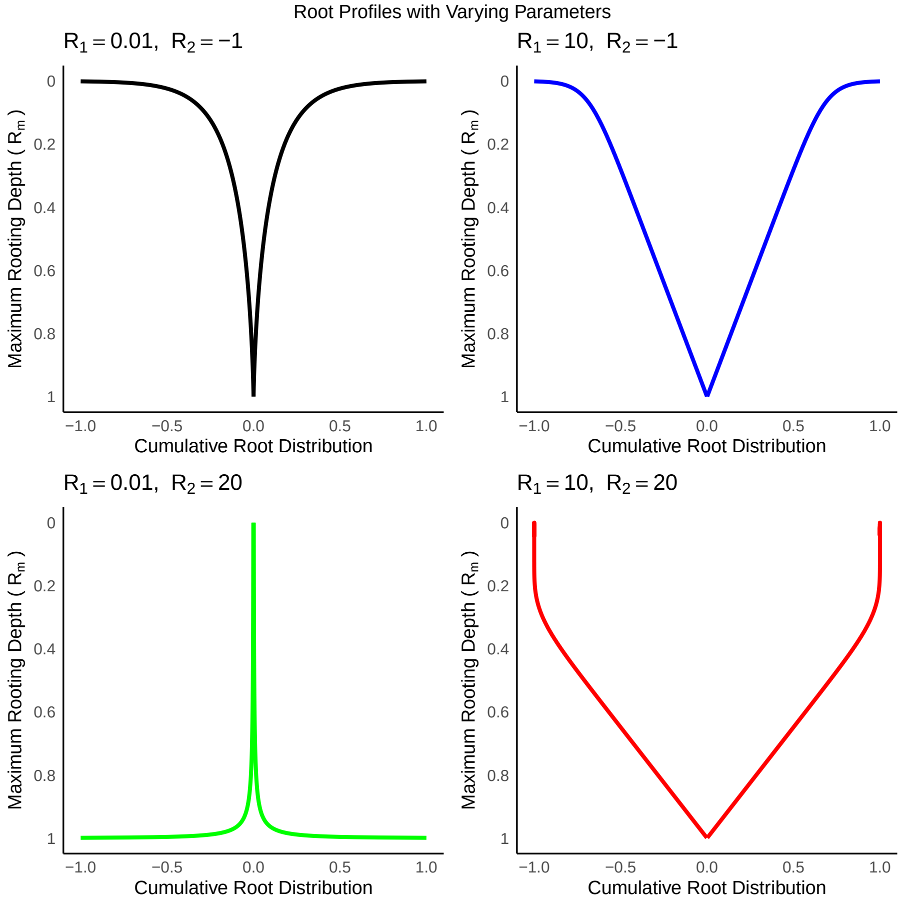

Figure S15: Extreme values of the rooting profiles permitted in our model based on the range of values for rooting traits presented in S9. These forms are broadly inline with forms presented in.[35] The top row displays shallow rooting profiles. Bottom left displays a tap root. Bottom right displays a rooting profile with roots more evenly apportioned across rooting depth.

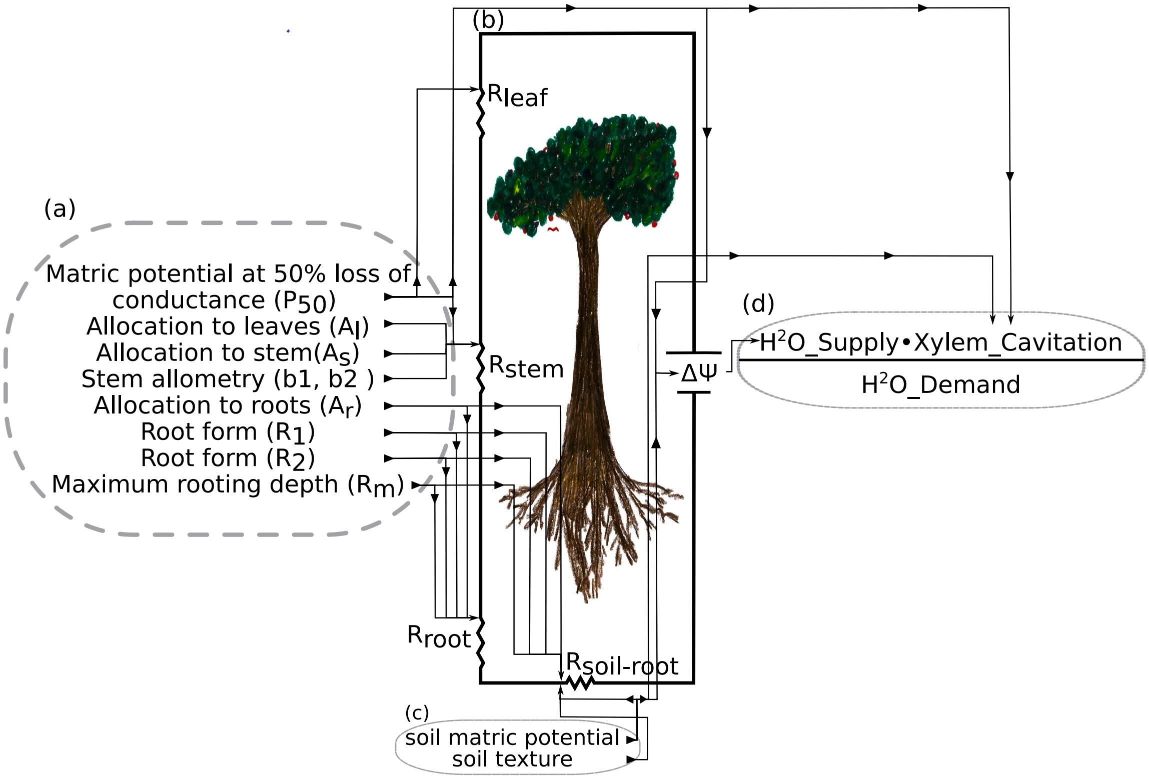

Figure S16: (a) Traits which can evolve and influence plant hydraulics in (b) and (d), (b) Ohm's law analogy of plant transpiration, $\Delta\Psi$ is the water potential difference between soil and leaf driving water flow while soil-root, root, stem, and leaf resistances mediate flow rates, (c) soil properties influencing (b) and (d), (d) Plant water availability G_w (eq.S54) is determined by the quotient of water supply E_{canopy}, which is modified by xylem cavitation G, and water demand E_p^t.

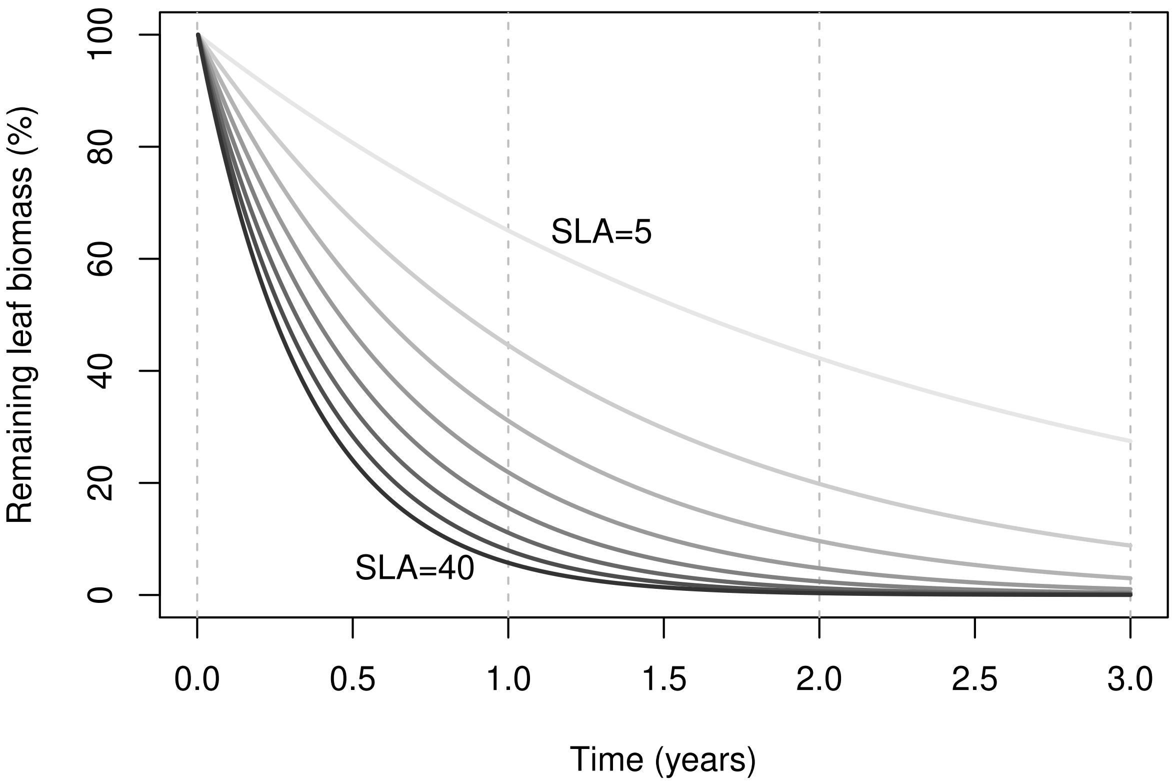

Figure S17: Leaf turnover as a function of SLA.

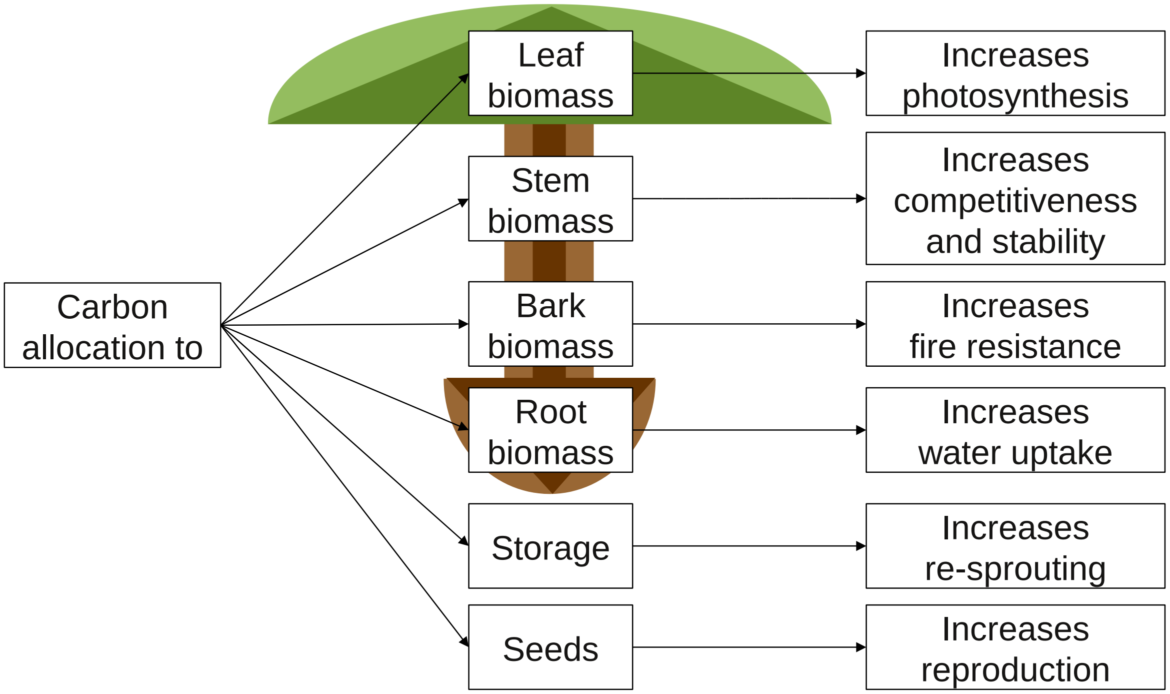

Figure S18: Biomass pools of single plants and benefits of carbon allocation to the biomass pools.

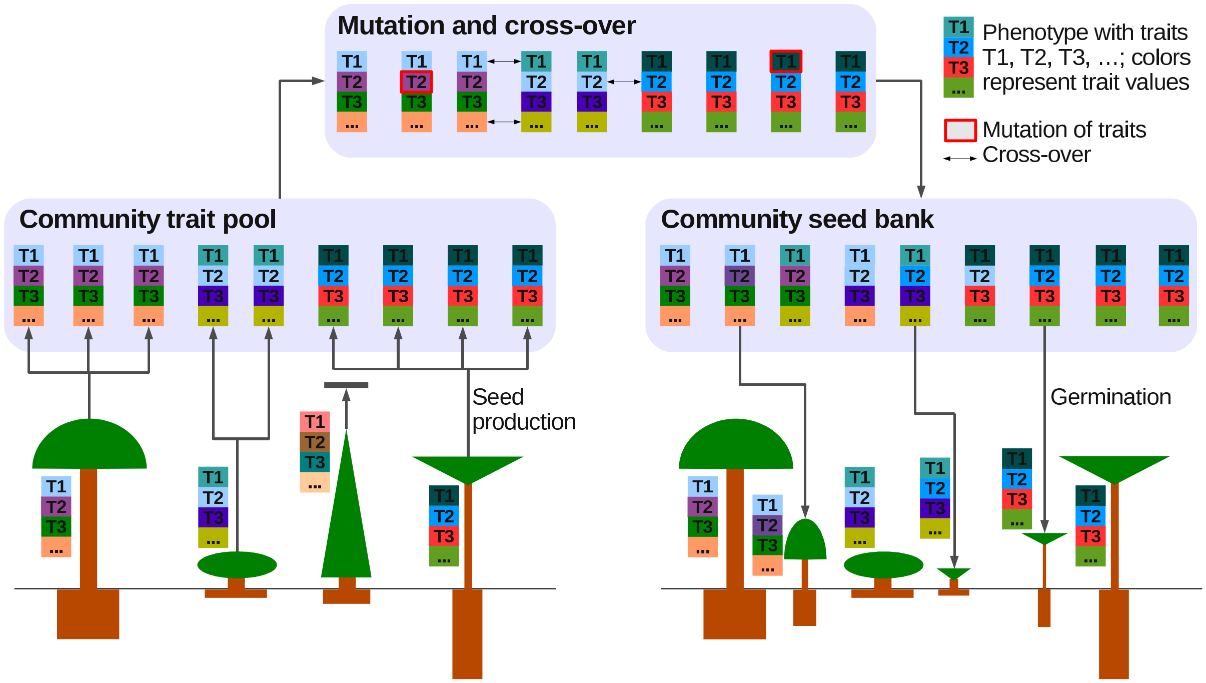

Figure S19: Scheme of the seed bank model. Plants produce seeds which are modified in the seed bank by mutation and cross-over.[14]

Table S9: Plant traits modified by the genetic optimisation algorithm. The columns 'Min' and 'Max' define the minimum and maximum possible trait values used for the genetic optimization algorithm for trees and C_4 grasses. Allocation traits sum to 1 (Mass Balance). Ideal min and max ranges (0-1),[65] some ranges were constrained to speed community assembly and time until equilibrium ecosystem dynamics are reached.

Name	Description	Trees		C_4 Grasses		Reference
		Min	Max	Min	Max	
P_{50}	Water potential at 50% loss of conductance	-3	-0.2	-3	-0.2	Continental/global SLA conversion[66,67]<br>Range (ca. 5-30)
A_R	Allocation to roots	0.2	0.4	0.2	0.8	Mass Balance<br>(Range 0-1)[65,68,69]
A_L	Allocation to leaves	0.35	0.5	0.25	0.5	Mass Balance<br>(Range 0-1)[65,68,69]
A_S	Allocation to stem	0.25	0.35	0	0	Mass Balance<br>[68,69]
A_B	Allocation to bark	0	0.05	0	0	Mass Balance<br>Up to ca. 20% of Stem Allocation[70]
A_T	Allocation to storage	0.1	0.4	0	0.4	Mass Balance<br>(Range 0-1)[65]
A_P	Allocation to reproduction	0.05	0.2	0.01	0.2	Mass Balance<br>Range (0-0.45)[71]
P_S	Phenology (rain/summergreen, evergreen)	0	1	0	1	Logical, Binary
P_E	Phenology (deciduous or evergreen)	0	1	0	0	Logical, Binary
T_{rd}	Rain threshold for plant dormancy	-3	-0.2	-3	-0.2	see S3.9<br>Range(T_{rd}) = Range(P_{50})
T_{lu}	Light threshold for plant activity	6	14	6	14	Phenomenological[54]<br>Radiation Range (MJ $m^{-2}day^{-1}$)
T_{ld}	Light threshold for plant dormancy	6	14	6	14	Phenomenological[54]<br>Radiation Range (MJ $m^{-2}day^{-1}$)
b_1	Parameter for height calculation	0.4	0.5	na	na	see S3.3.2<br>Following[32]
b_2	Parameter for height calculation	2.4	2.8	na	na	see S3.3.2<br>Following[32]
R_1	Parameter for the root form	0.01	10	0.01	10	Custom Function<br>see S3.6 Fig. S15
R_2	Parameter for the root form	-1	20	1	20	Custom Function<br>see S3.6 Fig. S15
R_m	Maximum rooting depth	1.0	10.0	1.0	1.0	Covers range of most<br>terrestrial biomes[72,73]
W	Seed weight	0.001	0.05	0.001	0.05	Approximate range of<br>mean across latitudes (g)[74]
C_2	Parameter for the canopy form	21	25	0	0	Following[31]
A_{TS}	Storage to stem allocation after fire	0.2	0.4	0	0	Phenomenological<br>ca. 25%[75]
A_{TL}	Storage to leaf allocation	0.6	0.9	0	1.0	Phenomenological<br>ca. 75%[75]

Table S10: Plant traits not modified by the genetic optimisation algorithm.

Name	Description	Trees	C_4 Grasses	Reference
M_C	Mortality: negative carbon balance	0.2	0.4	tuning parameter
M_B	Mortality: low height	0.05	na	tuning parameter
M_I	Mortality: mechanic instability	10	na	tuning parameter
T_C	Topkill parameter	4.3	0	[62]
T_H	Topkill parameter	5.003	0	[62]
T_I	Topkill parameter	0.004408	0	[62]
M	Ball Berry constant	9.0	4.0	[29]
B	Ball Berry constant	0.01	0.04	[29]
R_m	Maintenance respiration constant	0.015	0.025	[23,24]
R_g	Growth respiration constant	0.35	0.35	[76]
R_l	Respiration constant, leaves	0.01	0.01	[13]
R_s	Respiration constant, stem	0.01	0.01	[13]
R_r	Respiration constant, roots	0.01	0.01	[13]
R_n	Respiration constant	0.281	0.281	[76]
v_l	C:N ratio leaves	120	120	[13]
v_s	C:N ratio stem	150	120	[13]
v_r	C:N ratio roots	60	120	[13]
C_1	Parameter for the canopy form	10.0	na	following[31]

Table S11: Photosynthesis sub-model.

Name	Description	Value C_3	Value C_4	Units
A_{max}	Maximum light saturated photosynthesis	variable	variable	$\mu mol/m^2 s$
A_0	Gross photosynthetic rate	variable	variable	$\mu mol/m^2 s$
A_n	Net photosynthetic rate	variable	variable	$\mu mol/m^2 s$
A_0^b	Gross photosynthesis (bio-physical)	variable	variable	$\mu mol/m^2 s$
A_n^b	Net photosynthesis (bio-physical)	variable	variable	$\mu mol/m^2 s$
A_n^d	Net photosynthesis (diffusion)	variable	variable	$\mu mol/m^2 s$
V_{max}	Maximum carboxylation rate	variable	variable	$\mu mol/m^2 s$
A_R	Scaling factor for C_4 photosynthesis	1	39/90	unitless
A_S	Scaling factor for V_{max}	2	2	unitless
R_{mLs}	Constant leaf level respiration rate	0.82	1.36	$\mu mol/m^2 s$
c_a	Atmospheric partial pressure of CO_2	38.1	38.1	Pa
c_i	Internal CO_2 pressure (C_3 only)	$0.7c_a$	—	Pa
c_i	Bundle sheath value (C_4 only)	—	$8c_a$	Pa
K_c	Michaelis constant for CO_2	variable	variable	Pa
K_o	O_2 inhibition constant	variable	variable	Pa
τ	Fraction of RuBP to reaction of rubisco	variable	variable	prop
$f_{25}(T)$	Temperature function for K_c, K_o, τ	variable	variable	unitless
K_{25,K_c}	Constant for f_{25} for K_c	30	140	Pa
Q_{10,K_c}	Constant for f_{25} for K_c	2.1	2.1	Pa
K_{25,K_o}	Constant for f_{25} for K_o	30	34	Pa
Q_{10,K_o}	Constant for f_{25} for K_o	1.2	1.2	Pa
$K_{25,\tau}$	Constant for f_{25} for τ	2600	2600	Pa
$Q_{10,\tau}$	Constant for f_{25} for τ	0.57	0.67	Pa
O_i	Intercellular partial pressure of oxygen	21	21	kPa
Γ_*	CO_2 compensation point	variable	variable	Pa
J_c	Rubisco limited assimilation rate	variable	variable	$\mu mol/m^2 s$
J_e	Light limited assimilation rate	variable	variable	$\mu mol/m^2 s$
J_s	Transport limited assimilation rate for C_3	variable	—	$\mu mol/m^2 s$
J_p	CO_2 limited assimilation rate for C_4	—	variable	$\mu mol/m^2 s$
a	Leaf absorbtance of incident flux	0.86	0.80	unitless
α	Intrinsic quantum yield of photosynthesis	0.08	0.067	unitless
κ	Initial slope of response of CO_2	—	$0.7 \cdot 10^6$	$\mu mol/m^2 s$
V_{max}^c	Initial estimation for V_{max}	0.8	0.4	$\mu mol/m^2 s$
r	Respiration as fraction of V_{max}	0.015	0.025	prop
c_p	Specific heat of moist air	$1.013 \cdot 10^{-3}$	$1.013 \cdot 10^{-3}$	$MJ/kgdegC$
λ	Latent heat of air	2.45	2.45	MJ/kg

Table S12: Stomatal conductance sub-model.

Name	Description	Value C_3	Value C_4	Units
g_s	Leaf level stomatal conductance	variable	variable	$^{\mu mol}/_{m^2 s}$
m	Empirical parameter for g_s	9	4	unitless
b	Empirical parameter for g_s	0.01	0.04	$^{\mu mol}/_{m^2 s}$
g_b	Leaf level boundary layer conductance	variable	variable	m
c_s	Partial pressure of CO_2 at leaf surface	variable	variable	Pa
D_L	Characteristic leaf dimension	0.02	0.005	m
$\bar{H}$	Mean vegetation height	1.5	1.5	m
$u(z)$	Wind at height z from ground level	variable	variable	$^{m}/_{s}$
z_d	Displacement height	$0.86\bar{H}$	$0.86\bar{H}$	m
z_0	Roughness length	$0.06\bar{H}$	$0.06\bar{H}$	m
z_{ref}	Reference height	10	10	m

References

[1] New, M., Lister, D., Hulme, M. & Makin, I. A high-resolution data set of surface climate over global land areas. *Climate Research* **21**, 1–25 (2002).

[2] Giorgetta, M. A. *et al.* Climate and carbon cycle changes from 1850 to 2100 in MPI-ESM simulations for the Coupled Model Intercomparison Project phase 5. *Journal of Advances in Modeling Earth Systems* **5**, 572–597 (2013).

[3] Lehmann, C. E. R., Archibald, S. A., Hoffmann, W. A. & Bond, W. J. Deciphering the distribution of the savanna biome. *New Phytologist* **191**, 197–209 (2011).

[4] Bodegom, P. M. v., Douma, J. C. & Verheijen, L. M. A fully traits-based approach to modeling global vegetation distribution. *Proceedings of the National Academy of Sciences* **111**, 13733–13738 (2014).

[5] Butler, E. E. *et al.* Mapping local and global variability in plant trait distributions. *Proceedings of the National Academy of Sciences* **114**, E10937–E10946 (2017).

[6] Boonman, C. C. F. *et al.* Assessing the reliability of predicted plant trait distributions at the global scale. *Global Ecology and Biogeography* **29**, 1034–1051 (2020). _eprint: https://onlinelibrary.wiley.com/doi/pdf/10.1111/geb.13086.

[7] Madani, N. *et al.* Future global productivity will be affected by plant trait response to climate. *Scientific Reports* **8**, 2870 (2018).

[8] Moreno-Martínez, *et al.* A methodology to derive global maps of leaf traits using remote sensing and climate data. *Remote Sensing of Environment* **218**, 69–88 (2018).

[9] Schiller, C., Schmidtlein, S., Boonman, C., Moreno-Martínez, A. & Kattenborn, T. Deep learning and citizen science enable automated plant trait predictions from photographs. *Scientific Reports* **11**, 16395 (2021).

[10] Dong, N., Dechant, B., Wang, H., Wright, I. J. & Prentice, I. C. Global leaf-trait mapping based on optimality theory. *Global Ecology and Biogeography* **32**, 1152–1162 (2023).

[11] Langan, L., Higgins, S. I. & Scheiter, S. Climate-biomes, pedo-biomes or pyro-biomes: which world view explains the tropical forest–savanna boundary in South America? *Journal of Biogeography* **44**, 2319–2330 (2017).

[12] Langan, L. *Holism in plant biogeography - improving the representation of, and interactions between the biosphere, hydrosphere, atmosphere and pedosphere*. PhD Thesis, Institute of Physical Geography, Goethe University Frankfurt am Main, Germany (2019).

[13] Scheiter, S. & Higgins, S. I. Impacts of climate change on the vegetation of Africa: an adaptive dynamic vegetation modelling approach. *Global Change Biology* **15**, 2224–2246 (2009).

[14] Scheiter, S., Langan, L. & Higgins, S. I. Next-generation dynamic global vegetation models: learning from community ecology. *New Phytologist* **198**, 957–969 (2013).

[15] Sakschewski, B. *et al.* Leaf and stem economics spectra drive diversity of functional plant traits in a dynamic global vegetation model. *Global Change Biology* n/a–n/a (2015).

[16] Fisher, R. *et al.* Assessing uncertainties in a second-generation dynamic vegetation model caused by ecological scale limitations. *New Phytologist* **187**, 666–681 (2010).

[17] Clark, J. S. *et al.* Individual-scale variation, species-scale differences: inference needed to understand diversity. *Ecology Letters* **14**, 1273–1287 (2011).

[18] Sperry, J. S. & Love, D. M. What plant hydraulics can tell us about responses to climate-change droughts. *New Phytologist* 14–27 (2015).

[19] Sperry, J. S., Adler, F. R., Campbell, G. S. & Comstock, J. P. Limitation of plant water use by rhizosphere and xylem conductance: results from a model. *Plant, Cell and Environment* **21**, 347–359 (1998).

[20] Markesteijn, L., Poorter, L., Paz, H., Sack, L. & Bongers, F. Ecological differentiation in xylem cavitation resistance is associated with stem and leaf structural traits. *Plant, Cell & Environment* **34**, 137–148 (2011).

[21] Nachtergaele, F., van Velthuizen, H., Verelst, L. & Wiberg, D. Harmonised World Soil Database version 1.2, FAO, Rome and IIASA, Laxenburg, Austria (2012).

[22] Allen, R. G., Pereira, L. S., Raes, D. & Smith, M. *Crop evapotranspiration: Guidelines for computing crop water requirements* (Irrigation & Drainage, Paper 56, FAO, Rome, Italy, 1998).

[23] Collatz, G. J., Ball, J. T., Grivet, C. & Berry, J. A. Physiological and environmental regulation of stomatal conductance, photosynthesis and transpiration: a model that includes a laminar boundary layer. *Agriculture and Forest Meteorology* **54**, 107–136 (1991).

[24] Collatz, G., Ribas-Carbo, M. & Berry, J. Coupled Photosynthesis-Stomatal Conductance Model for Leaves of C_4 Plants. *Functional Plant Biol.* **19**, 519–538 (1992).

[25] Farquhar, G. D., Caemmerer, S. V. & Berry, J. A. A biochemical-model of photosynthetic CO_2 assimilation in leaves of C_3 species. *Planta* **149**, 78–90 (1980).

[26] Larcher, W. W. .-M. a. *Physiological plant ecology : ecophysiology and stress physiology of functional groups* (Springer, Berlin, 2003), 4th ed. edn.

[27] Woodward, F. I., Smith, T. M. & Emanuel, W. R. A global land primary productivity and phytogeography model. *Global Biogeochemical Cycles* **9**, 471–490 (1995).

[28] Woodward, F. I. & Smith, T. M. Global Photosynthesis and Stomatal Conductance: Modelling the Controls by Soil and Climate. *Advances in Botanical Research* **20**, 1–41 (1994).

[29] Ball, J. T., Woodrow, I. E. & Berry, J. A. Progress in Photosynthesis Research. 221–224 (Nijhoff, Dordrecht, 1987).

[30] Jones, H. G. *Plants and microclimate: a quantitative approach to environmental plant physiology* (2nd edn. Cambridge University Press, 1992).

[31] Strigul, N., Pristinski, D., Purves, D., Dushoff, J. & Pacala, S. Scaling from Trees to Forests: Tractable Macroscopic Equations for Forest Dynamics. *Ecological Monographs* **78**, 523–545 (2008).

[32] Niklas, K. J. & Spatz, H.-C. Worldwide correlations of mechanical properties and green wood density. *American Journal of Botany* **97**, 1587–1594 (2010).

[33] Craine, J. M. *et al.* Global diversity of drought tolerance and grassland climate-change resilience. *Nature Climate Change* **3**, 63–67 (2013).

[34] Tucker, S. S., Craine, J. M. & Nippert, J. B. Physiological drought tolerance and the structuring of tallgrass prairie assemblages. *Ecosphere* **2**, art48 (2011).

[35] Fan, Y., Miguez-Macho, G., Jobbágy, E. G., Jackson, R. B. & Otero-Casal, C. Hydrologic regulation of plant rooting depth. *Proceedings of the National Academy of Sciences* **114**, 10572–10577 (2017).

[36] Ostle, N. J. *et al.* Integrating plant-soil interactions into global carbon cycle models. *Journal of Ecology* **97**, 851–863 (2009).

[37] Nepstad, D. C., Tohver, I. M., Ray, D., Moutinho, P. & Cardinot, G. Mortality of large trees and lianas following experimental drought in an amazon forest. *Ecology* **88**, 2259–2269 (2007).

[38] da Costa, A. C. L. *et al.* Effect of 7 yr of experimental drought on vegetation dynamics and biomass storage of an eastern Amazonian rainforest. *The New phytologist* **187**, 579–591 (2010).

[39] Galbraith, D. *et al.* Multiple mechanisms of Amazonian forest biomass losses in three dynamic global vegetation models under climate change. *New Phytologist* **187**, 647–665 (2010).

[40] Powell, T. L. *et al.* Confronting model predictions of carbon fluxes with measurements of Amazon forests subjected to experimental drought. *New Phytologist* **200**, 350–365 (2013).

[41] McDowell, N. G. *et al.* Evaluating theories of drought-induced vegetation mortality using a multimodel–experiment framework. *New Phytologist* **200**, 304–321 (2013).

[42] Hickler, T., Prentice, I. C., Smith, B., Sykes, M. T. & Zaehle, S. Implementing plant hydraulic architecture within the LPJ Dynamic Global Vegetation Model. *Global Ecology and Biogeography* **15**, 567–577 (2006).

[43] Tyree, M. T. The Cohesion-Tension theory of sap ascent: current controversies. *Journal of Experimental Botany* **48**, 1753–1765 (1997).

[44] Meinzer, F. C., Clearwater, M. J. & Goldstein, G. Water transport in trees: current perspectives, new insights and some controversies. *Environmental and Experimental Botany* **45**, 239–262 (2001). 2072.

[45] Verhoef, A. & Egea, G. Modeling plant transpiration under limited soil water: Comparison of different plant and soil hydraulic parameterizations and preliminary implications for their use in land surface models. *Agricultural and Forest Meteorology* **191**, 22–32 (2014).

[46] Rawls, W. J., Brakensiek, D. L. & Saxtonn, K. E. Estimation of Soil Water Properties. *Transactions of the ASAE* **25**, 1316–1320 (1982).

[47] Saxton, K. E. & Rawls, W. J. Soil Water Characteristic Estimates by Texture and Organic Matter for Hydrologic Solutions. *Soil Science Society of America Journal* **70** (2006).

[48] Clapp, R. B. & Hornberger, G. M. Empirical equations for some soil hydraulic properties. *Water Resources Research* **14**, 601–604 (1978).

[49] Boone, A. *et al.* The Rhône-Aggregation Land Surface Scheme Intercomparison Project: An Overview. *Journal of Climate* **17**, 187–208 (2004).

[50] Fisher, R. A. *et al.* The response of an Eastern Amazonian rain forest to drought stress: results and modelling analyses from a throughfall exclusion experiment. *Global Change Biology* **13**, 2361–2378 (2007).

[51] Williams, M., Law, B. E., Anthoni, P. M. & Unsworth, M. H. Use of a simulation model and ecosystem flux data to examine carbon-water interactions in ponderosa pine. *Tree Physiology* **21**, 287–298 (2001).

[52] Tilman, D. *Plant Strategies and the Dynamics and Structure of Plant Communities* (Princeton University Press, 1988).

[53] Friedlingstein, P., Joel, G., Field, C. B. & Fung, I. Y. Toward an allocation scheme for global terrestrial carbon models. *Global Change Biology* **5**, 755–770 (1999).

[54] Lopes, A. P. *et al.* Leaf flush drives dry season green-up of the Central Amazon. *Remote Sensing of Environment* **182**, 90–98 (2016).

[55] Price, K. V., Storn, R. M. & A, L. J. *Differential Evolution - A Practical Approach to Global Optimization* (Springer-Verlag, 2005).

[56] R Development Core Team. *R: A Language and Environment for Statistical Computing* (R Foundation for Statistical Computing, Vienna, Austria, 2008). ISBN 3-900051-07-0.

[57] Mullen, K., Ardia, D., Gil, D., Windover, D. & Cline, J. *DEoptim: An R Package for Global Optimization by Differential Evolution.* (2009).

[58] Denslow, J. S. Tropical Rainforest Gaps and Tree Species Diversity. *Annual Review of Ecology and Systematics* **18**, 431–451 (1987).

[59] Pearson, T. R. H., Burslem, D. F. R. P., Mullins, C. E. & Dalling, J. W. Germination Ecology of Neotropical Pioneers: Interacting Effects of Environmental Conditions and Seed Size. *Ecology* **83**, 2798–2807 (2002).

[60] Marks, C. O. & Lechowicz, M. J. A holistic tree seedling model for the investigation of functional trait diversity. *Ecological Modelling* **193**, 141–181 (2006).

[61] Higgins, S. I., Bond, W. J., Trollope, W. S. W. & Williams, R. J. Physically motivated empirical models for the spread and intensity of grass fires. *International Journal of Wildland Fire* **17**, 595–601 (2008).

[62] Higgins, S. I., Bond, W. J. & Trollope, W. S. Fire, resprouting and variability: a recipe for grass-tree coexistence in savanna. *Journal of Ecology* **88**, 213–229 (2000).

[63] Hoffmann, W. A. *et al.* Tree topkill, not mortality, governs the dynamics of savanna-forest boundaries under frequent fire in central Brazil. *Ecology* **90**, 1326–1337 (2009).

[64] Hoffmann, W. A. *et al.* Ecological thresholds at the savanna-forest boundary: how plant traits, resources and fire govern the distribution of tropical biomes. *Ecology Letters* **15**, 759–768 (2012).

[65] Pavlick, R., Drewry, D. T., Bohn, K., Reu, B. & Kleidon, A. The Jena Diversity-Dynamic Global Vegetation Model (JeDi-DGVM): a diverse approach to representing terrestrial biogeography and biogeochemistry based on plant functional trade-offs. *Biogeosciences* **10**, 4137–4177 (2013).

[66] Fyllas, N. M. *et al.* Basin-wide variations in foliar properties of Amazonian forest: phylogeny, soils and climate. *Biogeosciences* **6**, 2677–2708 (2009).

[67] Bouchard, E. *et al.* Global patterns and environmental drivers of forest functional composition. *Global Ecology and Biogeography* **33**, 303–324 (2024).

[68] Chapin, F. S. *Principles of terrestrial ecosystem ecology* (Springer, New York ; London, 2002).

[69] Rius, B. F. *et al.* Higher functional diversity improves modeling of Amazon forest carbon storage. *Ecological Modelling* **481**, 110323 (2023).

[70] Neumann, M. & Lawes, M. J. Quantifying carbon in tree bark: The importance of bark morphology and tree size. *Methods in Ecology and Evolution* **12**, 646–654 (2021).

[71] Bazzaz, F. A., Ackerly, D. D. & Reekie, E. G. Reproductive allocation in plants. In *Seeds: the ecology of regeneration in plant communities*, 1–29 (CABI Wallingford UK, 2000).

[72] Canadell, J. *et al.* Maximum rooting depth of vegetation types at the global scale. *Oecologia* **108**, 583–595 (1996).

[73] Schenk, H. J. & Jackson, R. B. Rooting depths, lateral root spreads and below-ground/above-ground allometries of plants in water-limited ecosystems. *Journal of Ecology* **90**, 480–494 (2002).

[74] Moles, A. T. *et al.* Global patterns in seed size. *Global Ecology and Biogeography* **16**, 109–116 (2007).

[75] Schutz, A. E. N., Bond, W. J. & Cramer, M. D. Juggling carbon: allocation patterns of a dominant tree in a fire-prone savanna. *Oecologia* **160**, 235–246 (2009).

[76] Arora, V. K. Simulating energy and carbon fluxes over winter wheat using coupled land surface and terrestrial ecosystem models. *Agricultural and Forest Meteorology* **118**, 21–47 (2003).
