# Supplementary material for: Amazon forest resistance to drought is increased by diversity in hydraulic traits
Source: Nat Commun. 2025 Sep 9;16:8246. doi: 10.1038/s41467-025-63600-1 (PMC12420827; doi:10.1038/s41467-025-63600-1)
Supplement: Supplementary file 3 — Reporting Summary [file 41467_2025_63600_MOESM3_ESM.pdf]

nature portfolio

Corresponding author(s): Liam Langan

Last updated by author(s): 21.07.2025

Reporting Summary

Nature Portfolio wishes to improve the reproducibility of the work that we publish. This form provides structure for consistency and transparency in reporting. For further information on Nature Portfolio policies, see our Editorial Policies and the Editorial Policy Checklist.

Statistics

For all statistical analyses, confirm that the following items are present in the figure legend, table legend, main text, or Methods section.

n/a	Confirmed	
☐	☒	The exact sample size (*n*) for each experimental group/condition, given as a discrete number and unit of measurement
☐	☒	A statement on whether measurements were taken from distinct samples or whether the same sample was measured repeatedly
☐	☒	The statistical test(s) used AND whether they are one- or two-sided<br>*Only common tests should be described solely by name; describe more complex techniques in the Methods section.*
☐	☒	A description of all covariates tested
☐	☒	A description of any assumptions or corrections, such as tests of normality and adjustment for multiple comparisons
☐	☒	A full description of the statistical parameters including central tendency (e.g. means) or other basic estimates (e.g. regression coefficient) AND variation (e.g. standard deviation) or associated estimates of uncertainty (e.g. confidence intervals)
☐	☒	For null hypothesis testing, the test statistic (e.g. *F*, *t*, *r*) with confidence intervals, effect sizes, degrees of freedom and *P* value noted<br>*Give P values as exact values whenever suitable.*
☐	☒	For Bayesian analysis, information on the choice of priors and Markov chain Monte Carlo settings
☒	☐	For hierarchical and complex designs, identification of the appropriate level for tests and full reporting of outcomes
☒	☐	Estimates of effect sizes (e.g. Cohen's *d*, Pearson's *r*), indicating how they were calculated

Our web collection on statistics for biologists contains articles on many of the points above.

Software and code

Policy information about availability of computer code

Data collection	All data generated for this study was done using aDGVM2. The model has been previously published and fully described in Langan et al (2017) (https://doi.org/10.1111/jbi.13018).
Data analysis	All analysis was performed using R Statistical Software version 4.4.2.

For manuscripts utilizing custom algorithms or software that are central to the research but not yet described in published literature, software must be made available to editors and reviewers. We strongly encourage code deposition in a community repository (e.g. GitHub). See the Nature Portfolio guidelines for submitting code & software for further information.

Data

Policy information about availability of data

All manuscripts must include a data availability statement. This statement should provide the following information, where applicable:

- Accession codes, unique identifiers, or web links for publicly available datasets
- A description of any restrictions on data availability
- For clinical datasets or third party data, please ensure that the statement adheres to our policy

Processed data available at(https://doi.org/10.6084/m9.figshare.26232395). aDGVM2 (v1.0-LL) code available at (https://doi.org/10.5281/zenodo.16265490). Code embargoed until October first 2025 or until manuscript is published.

Research involving human participants, their data, or biological material

Policy information about studies with human participants or human data. See also policy information about sex, gender (identity/presentation), and sexual orientation and race, ethnicity and racism.

Reporting on sex and gender	NA
Reporting on race, ethnicity, or other socially relevant groupings	NA
Population characteristics	NA
Recruitment	NA
Ethics oversight	NA

Note that full information on the approval of the study protocol must also be provided in the manuscript.

Field-specific reporting

Please select the one below that is the best fit for your research. If you are not sure, read the appropriate sections before making your selection.

- [ ] Life sciences
- [ ] Behavioural & social sciences
- [x] Ecological, evolutionary & environmental sciences

For a reference copy of the document with all sections, see nature.com/documents/nr-reporting-summary-flat.pdf

Ecological, evolutionary & environmental sciences study design

All studies must disclose on these points even when the disclosure is negative.

Study description	This study uses a trait based vegetation model to examine whether diversity in plant functional traits affects tropicl forest responses to drought and climate change associated precipitation reductions.<br><br>For simulation results presented in Fig. 1 A, B drought treatments were applied and compared to observed changes in biomass at two tropical forest sites. These simulations were repeated 96 times for each site and mean responses were compared to observations. For simulation results presented in Fig. 1 C, D drought treatments were again applied and replicate simulations were run where the model was initialised with different numbers of functionally unique species (unique combination of trait-values). Each treatment containing (1,2,3,4,5,6,7,8,9,10,11,12,16,32,48,64,80,96) unique combinations of trait-values was repeated 96 times giving a total number of 1728 simulation runs for each site.<br><br>Future change simulations were run for tropical forest sites (1613 sites) across the Amazon basin for the time period up to 2100. Three sets of future change simulations were run with different diversity set-ups: 1) using the standard model parameterisiation; 2) using a paramaterisation where deciduous phenology which allows trees to flush or abscise leaves based on soil water potential was removed; 3) using a paramaterisation were deciduous and evergreen phenology which allows trees to flush or abscise their leaves based on soil water potential was removed. For each of 1-3, anomalies for RCP4.5 and 8.5 (2 levels) were applied. For each RCP anomaly, simulations were run with CO_2 held constant or CO_2 increasing in-line with the respective RCP scenario (2 levels). Thus each one of the 1613 sites was simulated 12 times (3 Diversity levels * 2 RCP anomalies * 2 CO_2 levels) giving a total of 19356 simulated grid-cells.
Research sample	The performance of the model when simulating drought was compared to observed percentage changes in biomass observed at two tropical forest sites where large-scale precipitation manipulations were performed. These observed field measurements represent the gold-standard in benchmarking model performance.<br><br>The simulated mean values of specific leaf area (SLA) for the study region was compared to a suite (7) of published observation based (1), data-driven machine learning based (5), and optimisation-based (1) products.
Sampling strategy	All simulation data was used for analysis. We did not perform field measurements.
Data collection	We did not carry out field measurements, presented results are out simulation model output.
Timing and spatial scale	Presented results are derived from simulations. Time period of presented results 1990-2100. Simulation were run for the Amazon basin.
Data exclusions	No data were excluded.
Reproducibility	All simulations have been re-run since initial submission owing to a mis-specification in one soil texture related parameter. The new results are essentially identical to the initially presented results. Thus, all attempts to repeat the simulation experiments were successful.

Randomization	Randomisation not relevant as the presented results are simulation model output, no field measurements were carried out.
Blinding	Blinding not relevant as the presented results are simulation model output, no field measurements were carried out.

Did the study involve field work? ☐ Yes ☒ No

Reporting for specific materials, systems and methods

We require information from authors about some types of materials, experimental systems and methods used in many studies. Here, indicate whether each material, system or method listed is relevant to your study. If you are not sure if a list item applies to your research, read the appropriate section before selecting a response.

Materials & experimental systems

n/a	Involved in the study
☒	☐ Antibodies
☒	☐ Eukaryotic cell lines
☒	☐ Palaeontology and archaeology
☒	☐ Animals and other organisms
☒	☐ Clinical data
☒	☐ Dual use research of concern
☒	☐ Plants

Methods

n/a	Involved in the study
☒	☐ ChIP-seq
☒	☐ Flow cytometry
☒	☐ MRI-based neuroimaging

Plants

Seed stocks	NA
Novel plant genotypes	NA
Authentication	NA
